# Nanostructure-Dependent Electrical Conductivity Model Within the Framework of the Generalized Effective Medium Theory Applied to Poly(3-hexyl)thiophene Thin Films

**DOI:** 10.3390/polym16223227

**Published:** 2024-11-20

**Authors:** Henryk Bednarski, Ayman A. A. Ismail, Marcin Godzierz, Andrzej Marcinkowski, Muhammad Raheel Khan, Bożena Jarząbek, Barbara Hajduk, Pallavi Kumari

**Affiliations:** 1 Centre of Polymer and Carbon Materials, Polish Academy of Sciences, ul. M. Curie-Skłodowskiej 34, 41-819 Zabrze, Poland; aabdallah@polsl.pl (A.A.A.I.); mgodzierz@cmpw-pan.pl (M.G.); amarcinkowski@cmpw-pan.pl (A.M.); mukhan@polsl.pl (M.R.K.); bjarzabek@cmpw-pan.pl (B.J.); bhajduk@cmpw-pan.pl (B.H.); pkumari@cmpw-pan.pl (P.K.); 2 Joint Doctoral School, Silesian University of Technology, Akademicka 2a, 44-100 Gliwice, Poland

**Keywords:** organic semiconductors, electrical conductivity, thin films, P3HT

## Abstract

One of the key parameters characterizing the microstructure of a layer is its degree of order. It can be determined from optical studies or X-ray diffraction. However, both of these methods applied to the same layer may give different results because, for example, aggregates may contribute to the amorphous background in XRD studies, while in optical studies, they may already show order. Because we are usually interested in the optical and/or electrical properties of the layers, which in turn are closely related to their dielectric properties, determining the optical order of the layers is particularly important. In this work, the microstructure, optical properties and electrical conductivity of poly(3-hexyl)thiophene layers were investigated, and a model describing the electrical conductivity of these layers was proposed. The model is based on the generalized theory of the effective medium and uses the equation from the percolation theory of electrical conductivity for the effective medium of a mixture of two materials. The results indicate a key role of the aggregate size and limited conductivity of charge carriers, mainly due to structural imperfections that manifest themselves as an increase in the number of localized states visible in the subgap absorption near the optical absorption edge. The critical value of the order parameter and the corresponding values of the Urbach energy, excitonic linewidth and band gap energy are determined.

## 1. Introduction

Organic semiconductors (OSs) find important applications in organic electronics devices such as organic photovoltaics (OPVs), organic light-emitting diodes (OLEDs), organic field effect transistors (OFETs), sensors, etc., as components of their active layers [1,2,3,4]. When their easy processing and relatively low production costs are taken into account, they become even more attractive. For these reasons, OSs have inspired and stimulated enormous research efforts to determine the relationship between the microstructure of thin OS films and their properties, which have been summarized in various reviews and research articles, e.g., [5,6,7,8,9,10,11,12,13,14,15,16,17,18,19,20]. One of the most studied OSs is poly(3-hexyl)thiophene P3HT, which owes its popularity to the intensive research conducted over the past two decades on the optimization of the performance of photovoltaic cells based on the polymer/fullerene blend heterojunction (BHJ), e.g., [21,22,23,24,25]. Although other materials are currently used in BHJ-based OPVs, research using P3HT can still be considered as developing and of great interest, e.g., [26,27,28,29,30,31,32,33,34,35,36,37], and importantly, P3HT is often considered as a reference material in the study of the properties of new materials. The strong relationship between the microstructure and properties of thin polymer organic semiconductors provides opportunities to model their properties by modifying the microstructure of the layers. An important aspect of the microstructure of a layer is its degree of order. This can be assessed by methods such as optical studies, e.g., [33,37,38,39,40,41], and X-ray diffraction, e.g., [21,22,24,33,39,40]. However, these methods can give different results when applied to the same layer because X-ray diffraction may be affected by aggregates in the amorphous background, whereas optical studies can show order in these aggregates. Given that the optical and electrical properties of layers are closely linked to their dielectric properties, it is of great importance to understand the factors that link optical order to electrical properties. In such a task, electrical conductivity models that can be applied to layers of these materials are very helpful. It is commonly known that in OSs, intramolecular interactions are primarily covalent, while intermolecular interactions usually result from much weaker noncovalent interactions, the π–π interactions. Consequently, the conduction bands in organic crystals are much narrower compared to those in inorganic materials, and their band structure is easily disrupted by defects, resulting in localized states in the energy gap. The well-established theory of Conwell [41] and Mott [42] assumes that the conduction process in organic materials is largely governed by tunneling transitions of carriers between these localized states, assuming that the electronic wave functions of the localized states overlap sufficiently. This concept was further explored by Mott [42], who introduced the variable-range hopping theory (VRH). The transport properties of many OSs can be effectively explained by the VRH theory [43,44]. An analytical model of the electrical conductivity of doped amorphous OSs was developed by Li et al. [45] The microstructural determinants of charge transport in semicrystalline conjugated polymers were studied by Mollinger et al. [46] This approach gave an expression for microstructure-dependent mobility, which included crystalline, chain, and hopping mobility, the latter two depending on the critical percolation concentration of the crystalline phase [46]. More recently, Janus et al. [33] developed a model for charge carrier transport in ultrathin polymer films, which includes contributions from the polymer chain length, degree of aggregation, and crystallinity. In this model, the reciprocal of the effective mobility scales with the sum of the reciprocal hole mobility through the crystalline and amorphous phases [33]. Another approach to describe conductivity in PEDOT/PSS films was presented by Bednarski et al. [47] In their work, the optical properties of PEDOT/PSS thin films were linked to the intrinsic electrical conductivities using the generalized effective medium theory (GEMT). The PEDOT-to-PSS volume ratio was a mixing factor describing the content of randomly distributed highly conductive PEDOT nanocrystals in the weakly conductive PSS matrix. Here, the idea that the GEMT-based conductivity model for a mixture of two materials from [47] can be extended to P3HT films is developed. The challenge is to find an appropriate order parameter from optical measurements to enable the use of GEMT and to treat the P3HT layers as consisting of a mixture of an ordered, highly conductive phase with a disordered phase that is a poor conductor of charge carriers. To demonstrate the applicability of the GEMT-based conductivity model for P3HT thin films, two series of films were prepared from highly concentrated solutions with different microstructures, their structural and optical properties were studied, and their electrical conductivity was determined. As is shown, this model allows for the determination of the conductivity values of the highly conductive phase and the weakly conductive phase. This model can also be successfully used to optimize the electrical conductivity of organic semiconducting films due to modifying factors.

## 2. Materials and Methods

Poly(3-hexylthiophene-2,5-diyl) (P3HT) M106, with a molecular weight (Mw) of 34.1 kDa and a regioregularity (RR) of 94.7%, was supplied by Ossila. Chlorobenzene, with a purity of 99.5%, was purchased from POCH Gliwice, Poland. For solution preparation, P3HT M106 was used in quantities of 20 mg and 40 mg. The preparation of P3HT solutions involved dissolving the polymer in chlorobenzene to form two distinct concentrations: 20 mg/mL and 40 mg/mL. The solutions were subjected to continuous stirring for a duration of 24 h at an elevated temperature of 60 °C to ensure thorough dissolution. Post-stirring, the solutions were further homogenized using a roll mixer. Subsequent to homogenization, films of P3HT were deposited onto 1 mm thick microscope glass substrates. Prior to deposition, the glass substrates underwent a cleaning process in an ultrasonic bath, sequentially using deionized water and isopropanol, each for 10 min. The deposition was executed via a spin-coating method. For the 20 mg/mL solution, the spin-coating process was conducted at varying rates: 750 rpm, 1000 rpm, 1250 rpm, 1500 rpm, 3000 rpm, and 4500 rpm. Conversely, the 40 mg/mL solution was spin-coated at rates of 1250 rpm, 1500 rpm, 1575 rpm, 1725 rpm, 2000 rpm, 3500 rpm, 5000 rpm, and 7000 rpm. In all instances, the spinning time was one minute, and the acceleration time was 3 s. Following the spin-coating procedure, the films underwent an initial heating phase at 120 °C for 5 min to remove residual solvent. Subsequently, electrodes were applied to the film surfaces using silver paste. A final heating step at 65 °C for 5 min was performed to ensure optimal electrode attachment. In addition, two control groups of samples were prepared from solutions with concentrations of 10 and 30 mg/mL for XRD studies. The film preparation conditions are summarized in Table S1.

X-ray diffraction (XRD) measurements were performed on P3HT films deposited on glass substrates using a Bruker D8 Advance diffractometer equipped in an X-ray lamp with Cu anode (λ = 1.54 Å) working with a voltage difference of 40 KV and 40 mA current between electrodes.

Atomic force microscopy (AFM) surface imaging was performed using a Dimension ICON atomic force microscope equipped with a NanoScope V controller (BRUKER Corporation, Santa Barbara, CA, USA) operating in the soft tapping mode in an air atmosphere with a standard 125 mm long, with flexural stiffness of 42 N/m of a single crystal doped silicon cantilevers (Model PPP-NCH-10, NANOSENSORS). Images were obtained with a piezoelectric scanner with a nominal size of 85 × 85 mm. The micrographs were recorded using NanoScope Analysis 1.9 software (BRUKER Corporation, Santa Barbara, CA, USA).

Spectrophotometric measurements were performed using an ultraviolet–visible–near-infrared (UV–Vis–NIR) JASCO V-570 spectrometer (JASCO Corporation, Tokyo, Japan).

Ellipsometric measurements were performed using SENTECH SE850 spectrometer, Sentech, Krailling, Germany, and modeling using SpectraRay 3 software.

Electrical resistance measurements were performed using Keithley 1617, a single channel electrometer/high resistance meter using a dedicated high resistance measurement box.

## 3. Results

### 3.1. XRD

**Figure 1:**
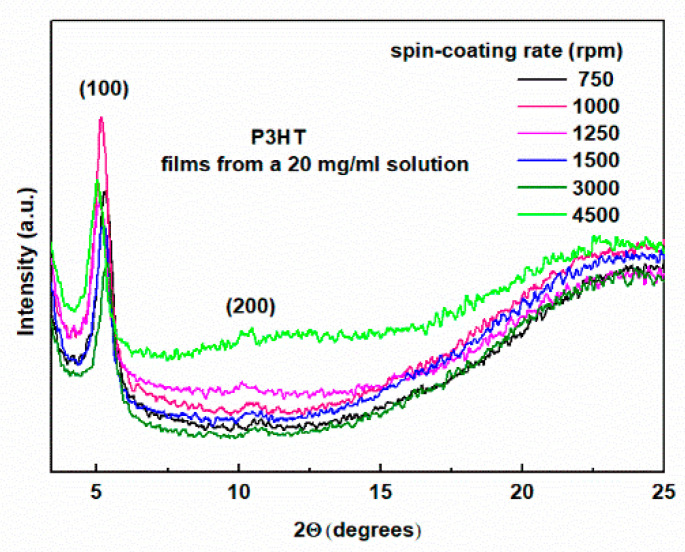
XRD patterns of P3HT thin films deposited from 20 mg/mL solution concentration with indicated spin-coating rates.

**Figure 2:**
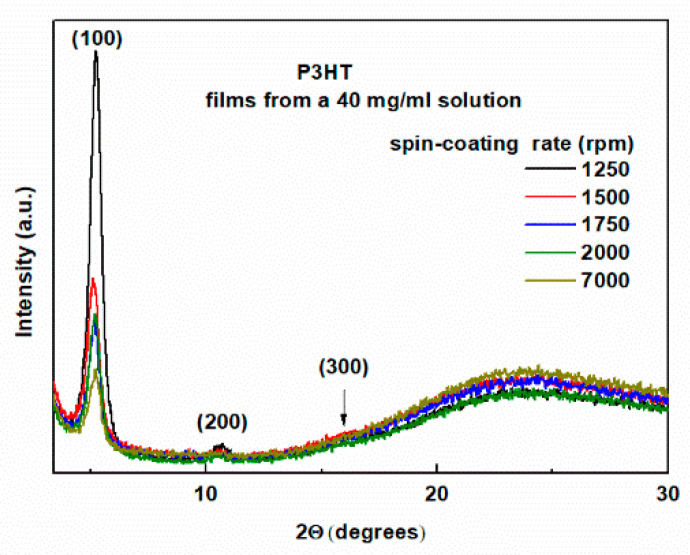
XRD patterns of P3HT thin films deposited from 40 mg/mL solution concentration with indicated spin-coating rates.

Measurements were performed in Bragg–Brentano geometry. In this experimental setup, X-ray diffractograms from lattice planes parallel to the film surface are visible. The XRD patterns of the tested samples are shown in Figure 1 and Figure 2 for the layers deposited on glass substrates from solutions with concentrations of 20 mg/mL and 40 mg/mL, respectively. As can be seen, the diffraction pattern of the layers is superimposed on the amorphous background originating from the glass substrate, visible as a large hump in the 2Θ angle range from 15 to 30 degrees. However, it does not obscure the intense (100) peak for 2Θ angles of about 5 degrees, clearly visible for all layers. This peak originates from the crystal lattice planes of crystallites separated by c.a. 16 Å and indicates the edge-on orientation of the P3HT chains [21,27,48].

We can also see the absence of the (020) peak for 2Θ of about 24 degrees, corresponding to the distance between the lattice planes of 3.8 Å, which would indicate the presence of crystallites containing polymer chains oriented face-to-face [21,25,48]. We can, therefore, conclude that the crystallites in the obtained layers show a preferred edge-on orientation of the P3HT chains.

**Figure 3:**
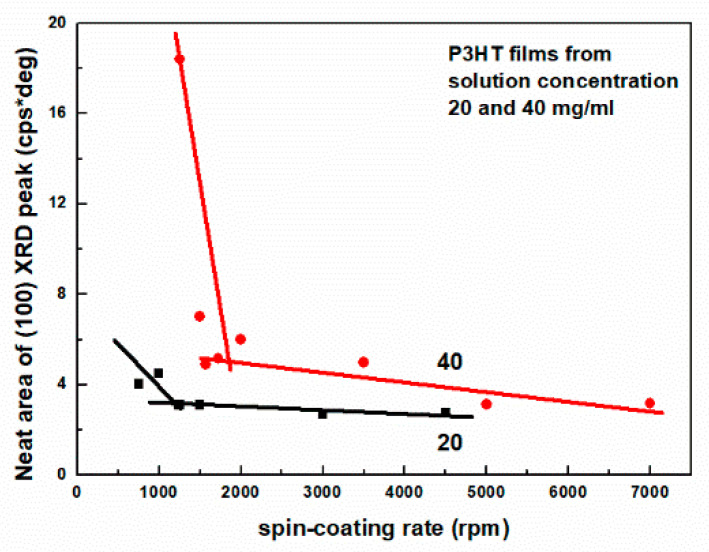
The neat area of (100) peak as a function spin-coating rate for P3HT thin films deposited from 20 and 40 mg/mL solution concentration.

This conclusion is further supported by the known fact that P3HT films prepared from chlorobenzene solution on a glass substrate crystallize in edge-on configurations [21]. Such orientation is favorable for conduction in the direction parallel to the layer surface, which coincides with the π–π stacking direction. Moving on to the discussion of the effect of the spin-coating rate on the morphology of the layers, we can see that with the increase of the spin speed, there is a clear decrease in the intensity of the (100) peak, in agreement with [27]. These changes are accompanied by a clear increase in the amorphousness of the layers, visible in the form of growing broad diffraction bands superimposed on the glass substrate background. This tendency is clearly visible in both of these figures. The difference is that for the layers obtained from the 20 mg/mL solution, these changes are limited to 2Θ of about 25 degrees. On the other hand, these changes for the layers shown in Figure 3 cover a larger range and are visible for 2Θ reaching 30 degrees. Importantly, such behavior was not observed in [27]. More detailed information is provided by the quantitative analysis of the (100) peak, the results of which are presented in Table 1 and Table 2 for the films from 20 mg/mL and 40 mg/mL solutions, respectively. As can be seen, the position of the (100) peak is within the range of 2Θ angles from 5.02 to 5.38 degrees for all the films. This corresponds to inter-planar spacings of 17.6 to 16.4 Å, and this parameter does not correlate with the spin rate. In contrast, the half-widths of this peak range from 0.480 to 0.548 degrees. However, the FWHM values for the films deposited from a 20 mg/mL solution are smaller than those obtained from a 40 mg/mL solution.

The crystallite size value is related to the FWHM because, according to the Scherrer formula used to determine it, it is inversely proportional to the FWHM. As can be seen from Table 1 and Table 2, the crystallite size ranges from 16.1 to 18.2 nm. As expected, the crystallite sizes for the films shown in Table 2 are larger than those shown in Table 2, which is consistent with their FWHM value. Finally, we note that the area of the (100) peak shows a clear correlation with the spin-coating rate. Namely, with increasing spin-coating rate, ω, the area of this peak gradually decreases. This is shown in Figure 3. As can be seen, these datasets cannot be well described by single linear fits. In fact, to describe one specific dataset, two lines with clearly different slopes are needed. Moreover, as can be seen, there are very narrow ranges of spinning rates for which films with good crystallinity can be obtained. We also note that the area of the (100) peak decreases rapidly with increasing spin speed during film deposition, and the crystal size changes only slightly (than that measured in the direction perpendicular to the film surface). From this fact, we conclude that the number of crystals in these layers decreases, or more precisely, the number of aggregates with long-range order decreases. 

To check the repeatability of the results, two additional groups of samples were prepared from solutions with concentrations of 10 and 30 mg/mL. The XRD patterns for these layers are shown in Figure S1a,b. The comparison shows that these results are fully consistent with the results presented in Figure 1 and Figure 2.

### 3.2. AFM

**Figure 4:**
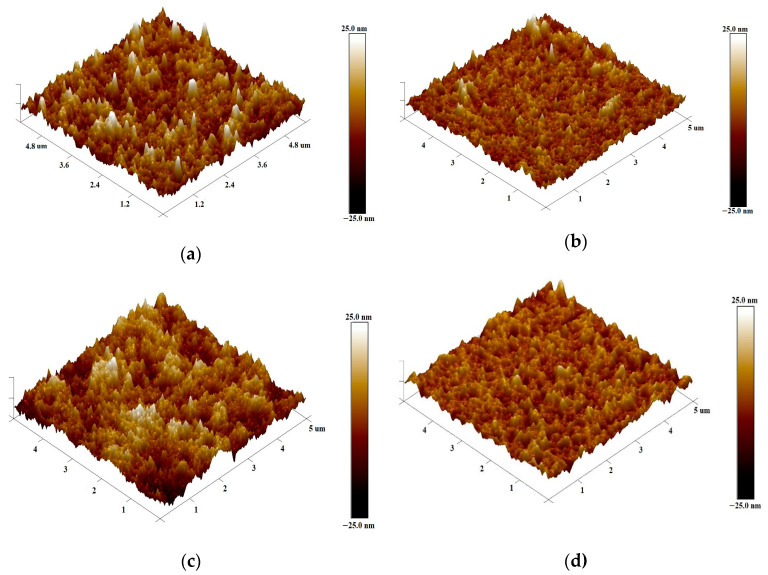
Three-dimensional surface AFM images of P3HT films deposited with spin-coating rates: (**a**) 750 rpm from 20 mg/mL solution; (**b**) 4500 rpm from 20 mg/mL solution; (**c**) 1250 rpm from 40 mg/mL solution; (**d**) 7000 rpm from 40 mg/mL solution.

The surface morphology of the layers was determined by atomic force microscopy (AFM). Due to the relatively large number of samples studied, surface images on a scale of 5 μm × 5 μm are shown in Figures S2a–h and S3a–f in the Supplementary Materials (SM). Here, in Figure 4, a comparison of surface images for only the thickest and thinnest layers is presented.

**Figure 5:**
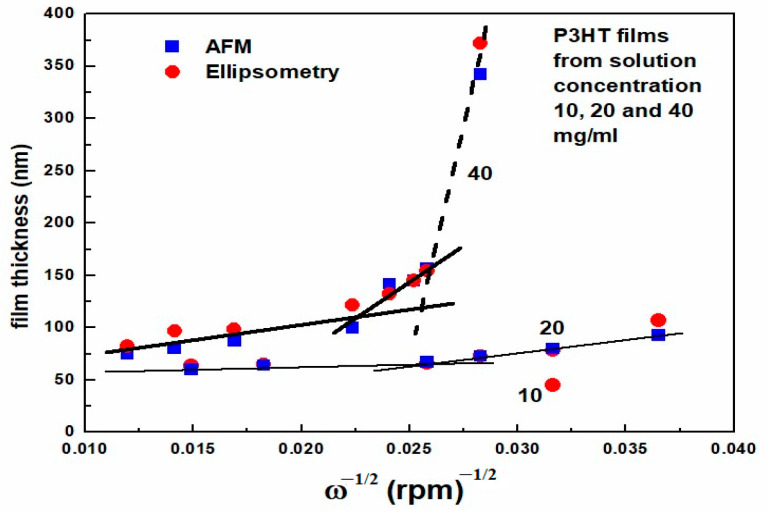
Thickness of the films determined in AFM and ellipsometry studies as a function of ω^−1/2^.

As can be seen, the layers obtained at low spin speed have a rougher surface with significantly sharper peaks. This is reflected in the value of the surface roughness parameter Rq, which shows a gradual decrease with increasing spin-coating speed of the layers, as shown in Figure S4. As can be seen, the linear fit for the layers deposited from 20 and 40 mg/mL solutions describes the results well in the whole ω range. The thickest layers were characterized by the highest surface roughness of 6 and 4.6 nm, while the thinnest layers were characterized by the lowest surface roughness of 4 and 3 nm. In turn, the thickness of the layers as a function of ω^−1/2^ is shown in Figure 5. Based on the spin-coating theory, such a graph is expected to be linear [49]. As can be seen, only the thickest layer obtained from a 40 mg/mL solution does not satisfy this relationship.

### 3.3. Optical Measurements

Optical studies of the layers included measurements of their transmission in the visible range and ellipsometric measurements in the UV–VIS range. Because the knowledge of the layer thickness is important in the presented studies, it was additionally determined based on ellipsometric measurements using the Cauchy optical model in the low absorption range, i.e., below the absorption edge. A comparison of the results of the layer thicknesses obtained by both methods is presented in Figure 5. As can be seen, these values agree well. In the further part of this work, we will use the values determined from AFM, in particular, to determine the absorption coefficients from the measured transmittances and to calculate the electrical conductivity of the layers from the measured resistance.

#### VIS Absorption Spectra Interpretation

The interpretation of the results includes the determination of the energy gap, Eg, the Urbach energy Eu, the exciton bandwidth W and the exciton linewidth σ. The methods for determining the values of these physical quantities are well-known and widely described in the literature, e.g., see references [50,51] for the determination of Eg and Eu and references [52,53,54] for W and σ and references therein. The transmittance of the studied films deposited from solutions with concentrations of 20 and 40 mg/mL are shown in Figures S5a and S5b, respectively. In general, the absorption spectra show the typical P3HT thin film shape of absorption bands. A distinct exciton peak at photon energy c.a. 2.05 eV and accompanying vibronic band maxima are visible.

However, significant differences can be seen in the results for the series of films deposited with different spin-coating rates, which become apparent near the optical absorption band edge. Namely, the films deposited with the lowest spin-coating rates have a steeper absorption edge. In the following, we will describe these differences in more detail.

**Figure 6:**
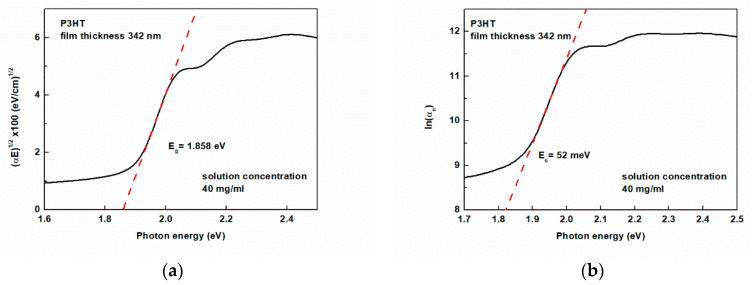
Example analysis of the absorption spectrum for a 342 nm thick layer to determine the values of (**a**) the band gap energy Eg; (**b**) the Urbach energy Eu.

**Figure 7:**
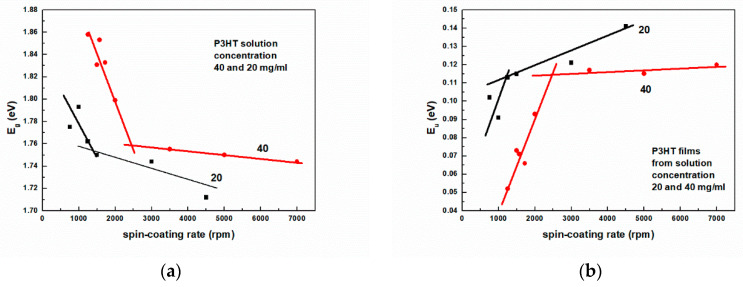
Dependences of (**a**) the energy gap Eg; (**b**) the Urbach energy Eu on the spin-coating rate.

To quantitatively describe these differences, we have determined values of all characteristic energetic quantities, i.e., Eg, Eu, W, and s. Exemplary results of analysis of the absorption spectrum for determining these quantities are shown in Figure 6a,b, whereas plots of Eg and Eu, as a function of spin-coating rate, are shown in Figure 7a and Figure 7b, respectively. As can be seen, two straight lines are needed to describe the data well in the whole range of spin-coating rates. With increasing rates, the Urbach energy increases from c.a. 55 to 140 meV, and the Eg value of the layers decreases from 1.858 to 1.712 eV. The increase in Urbach energy indicates an increase in the number of defects in the form of localized low-energy states located below the optical absorption edge.

**Figure 8:**
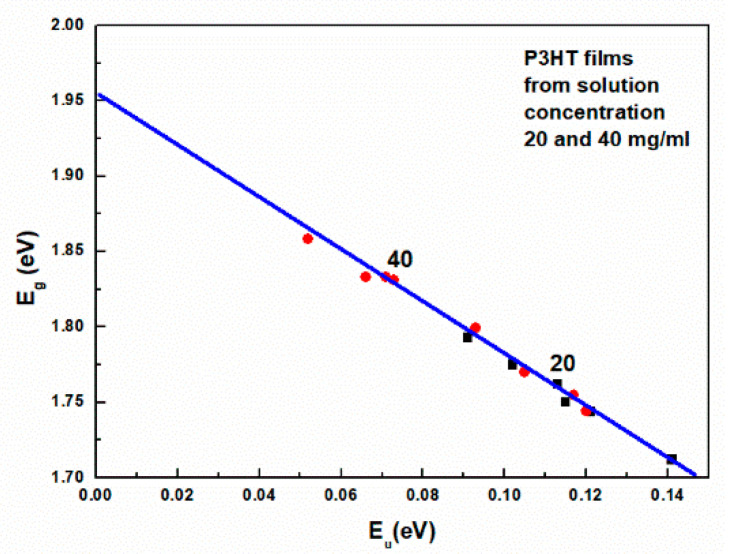
The band gap energy Eg is a function of the Urbach energy Eu.

The presence of these states is visible in the form of an exponentially decaying so-called Urbach tail, which is visible for energies higher than Eg. The observed decrease in Eg with increasing spin-coating rates has the same cause and results from the mutual correlation of both these quantities. Indeed, this is clearly visible in Figure 8, which shows Eg as a function of Urbach energy. As a test of the correctness of the analysis, one can read from this figure the extrapolated value of Eg = 1.96 eV for the layer with zero Urbach energy. This value should be close to the HOMO-LUMO energy difference.

We now turn to the interpretation of absorption spectra based on the theoretical work of Clark et al. [52] and Spano et al. [53,54] for aggregated polymer chain fragments in the weak interaction approximation [51]. As can be seen from the results of these works, the excitonic bandwidth can be determined from the ratio of the amplitudes of the first two absorption peaks.

**Figure 9:**
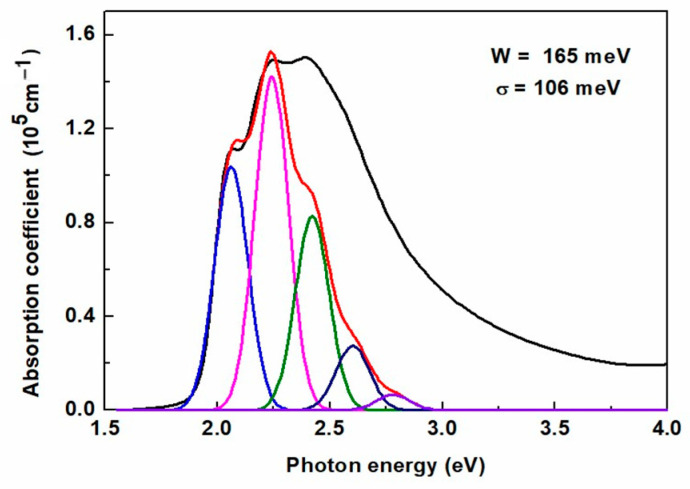
Example analysis of the absorption spectrum for a 342 nm thick P3HT film to determine the values of the exciton bandwidth W and the exciton linewidth σ.

More precisely, this theory allows calculating the absorption spectrum of aggregates as a sum of vibronic bands having the shape of Gaussian lines with the same broadening σ, also referred to as exciton line width. Compared to the experimental data, this approach gives an estimate not only of the values of W and σ but also of the percentage share of aggregates in the layer [52]. An example of applying this approach to the absorption coefficient of the thickest layer is shown in Figure 9, and the aggregate contribution is indicated by the red contour. As can be seen, the fit is not perfect. The amplitudes of the first two peaks are slightly too high. However, considering that only two parameters are fitted to a part of the spectrum, and W is the ratio of the amplitudes of the two peaks, this discrepancy is acceptable.

**Figure 10:**
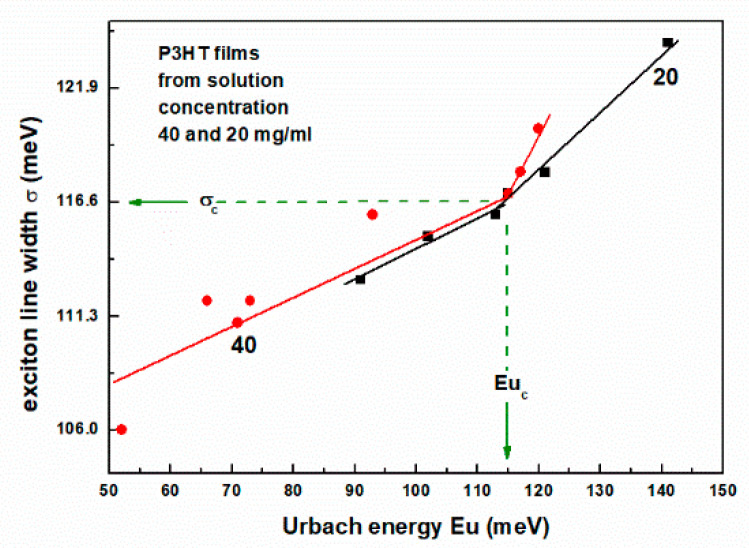
Dependences of the exciton linewidth σ. on the Urbach energy.

Plots of the exciton linewidth σ, from such fits, as a function of the Urbach energy, are shown in Figure 10. As can be seen, a clear change in slope occurs at the characteristic value of σ_c_ and Eu_c_, as indicated in Figure 10.

To go further, it is necessary to analyze in more detail the changes in the absorption spectrum of the films as a function of the spin-coating rate. The normalized absorption spectra of films obtained from solutions with concentrations of 20 and 40 mg/mL are shown in Figures S6a and S6b, respectively. The normalization was performed at a photon energy of 2.25 eV, corresponding to the second maximum of the vibronic band, using the film thickness as a tuning parameter. A comparison of the absorption spectra of the films deposited at different spin-coating rates shows that the absorption edge slope decreases with increasing spin-coating rate. It should be noted that the values of such quantities as Eu, W, and *σ*, determined from the absorption spectra, are independent of the film thickness. In other words, the accuracy of the determination of the film thickness does not affect the accuracy of their determination. It was mentioned above that the approach developed by Clark et al. [52] also allows us to determine the percentage of aggregates in the layer. However, when analyzing the results obtained from multiple layers, it should be taken into account that the density of aggregates in the layers may be different. For this reason, to explain our results, we will look for a more appropriate parameter. As it follows from the work of Spano [53], the exciton linewidth *σ* is a measure of disorder, and the value of this parameter is closely related to the number of molecules, *N*, forming the aggregate. In the approximation of spatially uncorrelated site disorder for *σ*, the following relation holds [53]:

(1) $$\sigma_{N-aggregate}=\sqrt{\frac{3}{2(N+1)}}\sigma_{molecule},$$

where $\sigma_{molecule}$ is the value of σ corresponding to a single molecule. Assuming the completely natural assumption that $\sigma_{molecule}$ has the same value for all layers studied, and that the value of $\sigma_{N-aggregate}$ is equal to the experimentally determined value of *σ* for a given layer, the corresponding values of *N* were determined from Equation (1). For this purpose, 11 quotients of *σ* were created from the experimentally determined values. Eight of these values were used to determine the values of $\sigma_{molecule}$ and the corresponding *N* numbers. The remaining three were used as control values. A surprisingly good agreement of c.a. 2 percent was obtained for the integer *N* values ranging from 21 to 30 molecules in the aggregate in the films studied and for $\sigma_{molecule}$ = 0.482. The details of this analysis are presented in the Supplementary Materials. It is worth noting that removing the restriction of *N* to integers can only improve the agreement. The analysis shows that an increase in the number of defects, and therefore in the Urbach energy values, should be associated with a decrease in the number of molecules in the aggregates.

**Figure 11:**
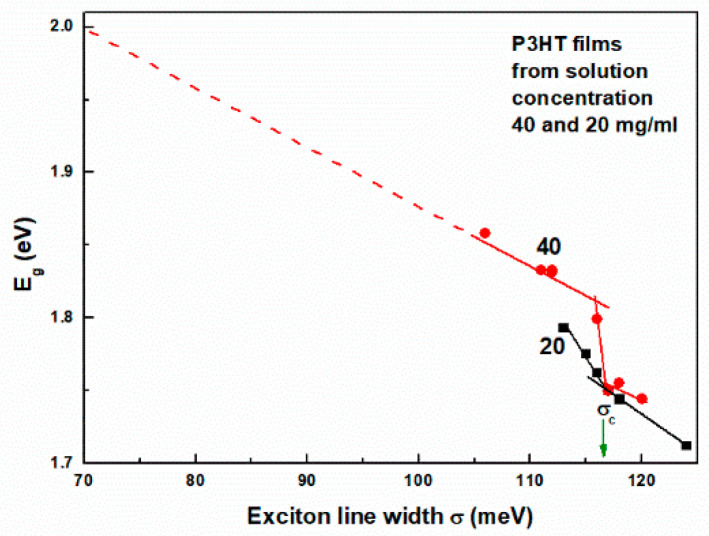
Band gap energy as a function of the exciton linewidth s.

Therefore, from the relation given by Equation (1), it follows that normalized *N* can be a good order parameter. Because Eg is linearly related to the Urbach energy, the normalization of *N* can be performed based on the extrapolated value of *σ* for Eg = 2 eV obtained from the plot of Eg versus *σ*, as shown in Figure 11. Now, again, using Equation (1), the number of molecules in such an aggregate can be determined to be *N(σ’)* = 70. In this way, we can define the order parameter as the ratio *N*(σ) to *N*(σ’).

### 3.4. Electrical Conductivity

As mentioned in the introduction to the description of the electrical conductivity of P3HT layers, we intend to use a model based on the generalized effective medium theory (GEMT). In this model, the equation from the percolation theory of electrical conductivity is applied to the effective medium formed by a mixture of two materials, which are good and bad electrical conductors. In [47], GEMT was successfully applied to describe the composition-dependent specific electrical conductivity of PEDOT/PSS. GEMT relates the specific electrical conductivities of the conductive inclusion material (*Σ*_c_) and the more resistive matrix material (*Σ*_r_) to the conductivity of the composite medium (*Σ*_m_) by the following equation [47,55,56]:

(2) $$\frac{\left(1-x)(\Sigma_{r}^{1/w}-\Sigma_{m}^{1/w}\right)}{\Sigma_{r}^{1/w}+\frac{1-x_{c}}{x_{c}}\Sigma_{m}^{1/w}}+\frac{x(\Sigma_{c}^{1/w}-\Sigma_{m}^{1/w})}{\Sigma_{c}^{1/w}+\frac{1-x_{c}}{x_{c}}\Sigma_{m}^{1/w}}=0$$

where *x* is the volume fraction of the conductive phase, *x*_c_ is the (critical) percolation volume fraction of the inclusion material, and the model parameter *w* denotes the critical exponent [47,55,56]. Thus, in accordance with the discussion presented above, the P3HT layers will be considered as consisting of a mixture of aggregates with different N numbers. It is obvious that good electrical conductors are those with appropriately large values of *N*, and bad ones—those with small values of *N*. As the order parameter, we take *x* = *N*(*σ*)/*N*(*σ*’). Equation (2) allows, among other things, to determine the average conductivity of aggregates that are well-conducting and those that are poorly conducting. It should be emphasized here that the critical value of the excitonic linewidth *σ*_c_, and therefore *N*_c_, has already been determined above based solely on optical measurements.

**Figure 12:**
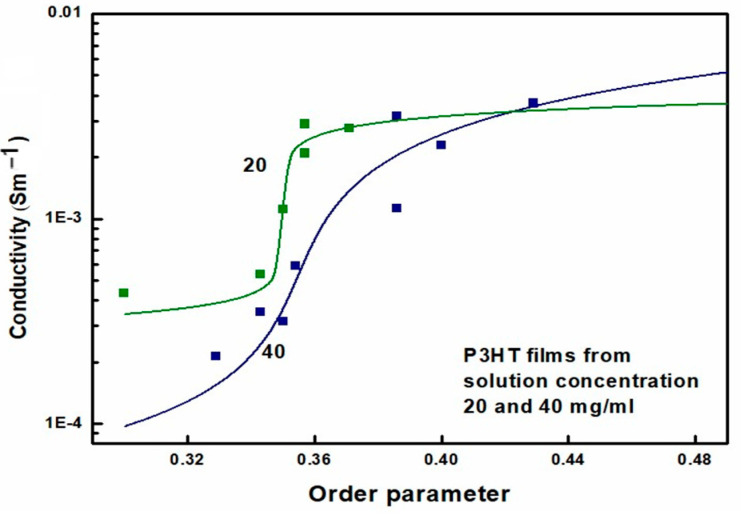
Conductivity of P3HT films as a function of the order parameter.

Electrical conductivity measurements were performed on all tested films with two silver paste contacts. The average distance between the contacts was 1 mm, and their width was 3 mm; therefore, the geometric factor was assumed to be equal to 3. Due to the high resistance of the layers, the two-probe measurement is sufficiently accurate. The specific resistance of the layers was determined from the current-voltage characteristics using the layer thicknesses determined from AFM studies. The final results of applying Equation (2) to the experimentally determined electrical conductivity of P3HT layers are shown in Figure 12. In this figure, the lines represent a 4-parameter fit of Equation (2) to the experimental data. Three of these parameters have a clear physical interpretation. Namely, the values of *Σ*_c_ and *Σ*_r_ are the extrapolated values of *Σ* for the limiting values of *x*, while *x*_c_ is the value of *x* at the onset of percolation. The values of *x*_c_ obtained from GEMT, i.e., Equation (2), are 0.35 and 0.355 for P3HT films obtained from 20 and 40 mg/mL solutions, respectively. In comparison, the critical value of the order parameter determined from optical measurements ranges from 0.343 to 0.357. Thus, the agreement of the critical value of *x*_c_ determined by both methods is good.

The crystallinity of OS thin films may depend on many factors. For example, the molar mass of the polymer and its regio-regularity, the type of solvent used, or the engineering of solutions such as the addition of antisolvents, etc. The electrical conductivity model developed here can be useful in studying the influence of these factors on the electrical conductivity of the OS films. In particular, it can be helpful in the work related to the transformation towards a green and digital economy because the use of non-halogenated solvents for environmental protection is crucial.

## 4. Discussion

In Clark’s seminal article on the analysis of absorption spectra of polythiophene films [50], a method for determining the percentage of aggregates was also presented. An important element of this description was the analysis of the changes in the absorption spectrum of the polymer solution during heating. During this process, the percentage of aggregates decreased at the expense of the increase in the amorphous fraction. This method can be used to determine the percentage of aggregates for a series of layers obtained for different deposition conditions. However, it should be noted that the number of aggregates in the layers can also change with the change in deposition conditions. For this reason, in this work, it was decided to look for another parameter describing the order in the layers. The average aggregate size, or more precisely, the number of molecules forming such an average aggregate, appeared to be a good alternative.

In this paper, the application of the generalized effective medium theory (GEMT) to the description of the electrical conductivity in P3HT films is presented. To achieve this, it was necessary to extend GEMT from a composition-dependent to a nanostructure-dependent model. This allowed us to obtain new insights into the charge transport in these layers. First of all, the role of the aggregate size and its close relationship with defects, and thus its relationship with the Urbach energy and the energy gap have been addressed. This approach differs from the recently presented model of Janus et al., see Ref. [33], or the slightly earlier model of Mollinger et al., see Ref. [46], in the way the charge carrier transport is averaged over the molecular structure, crystallinity and aggregation. The obtained results here indicate a key role of the aggregate size and limited conductivity of charge carriers, mainly due to structural imperfections that manifest themselves as an increase in the number of localized states visible in the subgap absorption near the optical absorption edge. The critical value of the order parameter and the corresponding values of the Urbach energy, excitonic linewidth, and band gap energy were determined.

## Figures and Tables

**Table 1:** Parameter values determined from XRD diffractograms for films prepared from 20 mg/mL P3HT solution.

Spin-Coating Rate (rpm)	Peak Position 2Θ (°)	FWHM (°)	Neat Area (cps ^.^°)	Crystallite Size (nm)
750	5.31	0.51	4.04	17.3
1000	5.19	0.49	4.49	18.1
1250	5.13	0.51	3.10	17.3
1500	5.26	0.52	3.11	17.0
3000	5.36	0.49	2.68	18.0
4500	5.02	0.48	2.78	18.2

**Table 2:** Parameter values determined from XRD diffractograms for films prepared from 40 mg/mL P3HT solution.

Spin-Coating Rate (rpm)	Peak Position 2Θ (°)	FWHM (°)	Neat Area (cps °)	Crystallite Size (nm)
1250	5.24	0.517	18.41	17.1
1500	5.14	0.511	7.01	17.3
1575	5.31	0.523	4.88	16.4
1725	5.16	0.535	5.17	16.5
2000	5.19	0.538	5.99	16.4
3500	5.27	0.520	4.98	17.0
5000	5.38	0.522	3.12	16.9
7000	5.20	0.548	3.17	16.1

## Data Availability

Data are contained within the article and the Supplementary Materials.

## Supplementary Materials

The following supporting information can be downloaded at: https://www.mdpi.com/article/10.3390/polym16223227/s1, FigureS1: XRD patterns of P3HT films deposited from solutions with concentrations: (a) of 10 mg/mL and (b) 30 mg/mL solution concentration with indicated spin-coating rates. Figure S2: Three-dimensional surface AFM images of P3HT films deposited with indicated spin-coating rates from 20 mg/mL solution; Figure S3: Three-dimensional surface AFM images of P3HT films deposited with indicated spin-coating rates from 40 mg/mL solution; Figure S4: Surface roughness parameter Rq, as a function of spin-coating rate; Figure S5: The transmittance of the studied films deposited from solutions with concentrations of 20 mg/mL and 40 mg/mL; Figure S6: Normalized absorption spectra of P3HT thin films deposited from solutions with concentrations of 20 mg/mL and 40 mg/mL Table S1: Summary of films deposition conditions; Details of the number of molecules, *N*, forming the aggregate determination.

## References

[1] Zhang G., Xie C., You P., Li S. (2022). Introduction to Organic Electronic Devices.

[2] Park S., Takakuwa M., Fukuda K., Lee S., Yokota T., Someya T. (2024). Spiers Memorial Lecture: Challenges and prospects in organic photonics and electronics. Faraday Discuss..

[3] Forrest S.R. (2020). Organic Electronics: Foundations to Applications.

[4] Lee H., Jiang Z., Yokota T., Fukuda K., Park S., Someya T. (2021). Stretchable organic optoelectronic devices: Design of materials, structures, and applications. Mater. Sci. Eng. R Rep..

[5] Coropceanu V., Cornil J., da Silva Filho D.A., Olivier Y., Silbey R., Brédas J.L. (2007). Charge transport in organic semiconductors. Chem. Rev..

[6] Sirringhaus H., Brown P.J., Friend R.H., Nielsen M.M., Bechgaard K., Langeveld-Voss B.M.W., de Leeuw D.D. (1999). Two-dimensional charge transport in self-organized, high-mobility conjugated polymers. Nature.

[7] Diao Y., Shaw L., Bao Z., Mannsfeld S.C. (2014). Morphology control strategies for solution-processed organic semiconductor thin films. Energy Environ. Sci..

[8] Zhang C., Mahadevan S., Yuan J., Ho J.K.W., Gao Y., Liu W., Zhong H., Yan H., Zou Y., Tsang S. (2022). Unraveling Urbach Tail Effects in High-Performance Organic Photovoltaics: Dynamic vs Static Disorder. ACS Energy Lett..

[9] Khasbaatar A., Xu Z., Lee J.-H., Campillo-Alvarado G., Hwang C., Onusaitis B.N., Diao Y. (2023). From Solution to Thin Film: Molecular Assembly of π-Conjugated Systems and Impact on (Opto)electronic Properties. Chem. Rev..

[10] Qu T., Nan G., Ouyang Y., Bieketuerxun B., Yan X., Qi Y., Zhang Y. (2023). Structure–Property Relationship, Glass Transition, and Crystallization Behaviors of Conjugated Polymers. Polymers.

[11] Akai R., Oka K., Dekura S., Yoshimi K., Mori H., Nishi K.R., Saeki A., Tohnai N. (2023). Precise Control of the Molecular Arrangement of Organic Semiconductors for High Charge Carrier Mobility. J. Phys. Chem. Lett..

[12] Balzer D., Kassal I. (2023). Mechanism of delocalization-enhanced exciton transport in disordered organic semiconductors. J.Phys. Chem. Lett..

[13] Günes S., Neugebauer H., Sariciftci N.S. (2007). Conjugated polymer-based organic solar cells. Chem. Rev..

[14] Li G., Zhu R., Yang Y. (2012). Polymer solar cells. Nat. Photonics.

[15] Jin H., Kim K., Kim K., Park S., Shin E.Y., Heo J.W., Lee H., Baek S.W., Kim I.S., Ahn H. (2024). Development of degradable networked-organic semiconductors and effects on charge carrier mobility in organic thin-film transistors. J. Mater. Chem. C.

[16] Kim T.H., Kim J.H., Kang K. (2023). Molecular doping principles in organic electronics: Fundamentals and recent progress. Jpn. J. Appl. Phys..

[17] Morab S., Sundaram M.M., Pivrikas A. (2023). Influence of traps and Lorentz Force on charge transport in organic semiconductors. Materials.

[18] Fratini S., Nikolka M., Salleo A., Schweicher G., Sirringhaus H. (2020). Charge transport in high-mobility conjugated polymers and molecular semiconductors. Nat. Mater..

[19] Morab S., Sundaram M.M., Pivrikas A. (2023). Review on charge carrier transport in inorganic and organic semiconductors. Coatings.

[20] Aurelio M.A. (2023). *π*-Conjugated Materials: Here, There, and Everywhere. Chem. Mater..

[21] Brinkmann M. (2011). Structure and Morphology Control in Thin Films of Regioregular Poly(3-hexylthiophene). J. Polym. Sci. Part B Polym. Phys..

[22] Ludwigs S. (2014). P3HT Revisited—From Molecular Scale to Solar Cell Devices.

[23] Neusser D., Malacrida C., Kern M., Gross Y.M., van Slageren J., Ludwigs S. (2020). High Conductivities of Disordered P3HT Films by an Electrochemical Doping Strategy. Chem. Mater..

[24] Nádaždy V., Gmucova K., Nádaždy P., Siffalovic P., Vegso K., Jergel M., Schauer F., Majkova E. (2018). Thickness Effect on Structural Defect-Related Density of States and Crystallinity in P3HT Thin Films on ITO Substrates. J. Phys. Chem..

[25] Na J.Y., Kang B., Sin D.H., Cho K., Park Y.D. (2015). Understanding solidification of polythiophene thin films during spin-coating: Effects of spin-coating time and processing additives. Sci. Rep..

[26] Ni Y., Liu J., Han H., Yu Q., Yang L., Xu Z., Jiang C., Liu L., Xu W. (2024). Visualized in-sensor computing. Nat. Commun..

[27] Bhagat S., Berteau-Rainville M., Beram S., Salzmann I. (2024). Amorphous Poly (3-hexylthiophene)(P3HT) Thin Films by Vacuum Electrospray Deposition. Cryst. Growth Des..

[28] Saporiti C., Brambilla L., Fazzi D., Castiglioni C. (2024). Insights into the Structural and Vibrational Properties of Polaron in Doped Poly(3-alkyl-thiophene), P3HT. J. Phys. Chem. C..

[29] Molefe F.V., Mothudi B.M., Dhlamini M.S. (2024). The effect of deposition method and thickness dependence on the growth of P3HT for organic photovoltaic devices. Results Opt..

[30] Qu S., Ming C., Yao Q., Lu W., Zeng K., Shi W., Shi X., Uher C., Chen L. (2018). Understanding the Intrinsic Carrier Transport in Highly Oriented Poly(3-hexylthiophene): Effect of Side Chain Regioregularity. Polymers.

[31] Brey D., Burghardt I. (2024). Coherent Transient Localization Mechanism of Interchain Exciton Transport in Regioregular P3HT: A Quantum-Dynamical Study. J. Phys. Chem. Lett..

[32] Kumari P., Hajduk B., Jarka P., Bednarski H., Janeczek H., Łapkowski M., Waśkiewicz S.A. (2024). Supramolecular Approach to Enhance the Optoelectronic Properties of P3HT-b-PEG Block Copolymer for Organic Field-Effect Transistors. ACS Omega.

[33] Janus K., Chlebosz D., Janke A., Goldeman W., Kiersnowski A. (2023). Contributions of Polymer Chain Length, Aggregation and Crystallinity Degrees in a Model of Charge Carrier Transport in Ultrathin Polymer Films. Macromolecules.

[34] Srivastava S., Rai A.K., Shrivastava M., Ramamurthy P.C. (2024). Sub-100 nm Patterning of P3HT with Enhanced OFET Device Performance by Dose Optimization of Electron Beam-Induced Cross-Linking. ACS Appl. Electron. Mater..

[35] Zhang L., Gregory S.A., Malinowski K.L., Atassi A., Freychet G., Losego M.D. (2024). Vapor Phase Infiltration of Titanium Oxide into P3HT to Create Organic-Inorganic Hybrid Photocatalysts. ACS Appl. Mater. Interfaces.

[36] Wu Y., Duong Q.M., Simafranca A.F., Salamat C.Z., Schwartz B.J., Tolbert S.H. (2024). Crystal Structure Control of the Energetics of Chemical Doping in Rub-Aligned P3HT Films. ACS Mater. Lett..

[37] Bednarski H., Hajduk B., Domański M., Jarząbek B., Nitschke P., Łaba K., Wanic A., Łapkowski M. (2018). Unveiling of polymer/fullerene blend films morphology by ellipsometrically determined optical order within polymer and fullerene phases. J. Polym. Sci. Part B Polym. Phys..

[38] Engmann S., Turkovic V., Denner P., Hoppe H., Gobsch G. (2012). Optical order of the polymer phase within polymer/fullerene blend films. J. Polym. Sci. Part B Polym. Phys..

[39] Noriega R., Rivnay J., Vandewal K., Koch F.P., Stingelin N., Smith P., Toney M.F., Salleo A. (2013). A general relationship between disorder, aggregation and charge transport in conjugated polymers. Nat. Mater..

[40] Kohn P., Huettner S., Komber H., Senkovskyy V., Tkachov R., Kiriy A., Friend H.R., Steiner U., Huck W.T.S., Sommer J.U. (2012). On the Role of Single Regiodefects and Polydispersity in Regioregular Poly(3-hexylthiophene): Defect Distribution, Synthesis of Defect-Free Chains, and a Simple Model for the Determination of Crystallinity. J. Am. Chem. Soc..

[41] Conwell E.M. (1956). Impurity Band Conduction in Germanium and Silicon. Phys. Rev..

[42] Mott N.F. (1956). On the transition to metallic conduction in semiconductors. Can. J. Phys..

[43] Kang S., Snyder G. (2017). Charge-transport model for conducting polymers. Nat. Mater..

[44] Poelking C., Daoulas K., Troisi A., Andrienko D., Ludwigs S. (2014). Morphology and Charge Transport in P3HT: A Theorist’s Perspective. P3HT Revisited—From Molecular Scale to Solar Cell Devices.

[45] Li L., Meller G., Kosina H. (2007). Analytical conductivity model for doped organic semiconductors. J. Appl. Phys..

[46] Mollinger S.A., Krajina B.A., Noriega R., Salleo A., Spakowitz A.J. (2015). Percolation, Tie-Molecules, and the Microstructural Determinants of Charge Transport in Semicrystalline Conjugated Polymers. ACS Macro Lett..

[47] Bednarski H., Hajduk B., Jurusik J., Jarząbek B., Domański M., Łaba K., Wanic A., Łapkowski M. (2016). The influence of PEDOT to PSS ratio on the optical properties of PEDOT: PSS thin solid films-insight from spectroscopic ellipsometry. Acta Phys. Pol. A.

[48] Kumari P., Hajduk B., Bednarski H., Jarka P., Janeczek H., Łapkowski M. (2023). Exploring the Influence of P3HT on PTCA Crystallization and Phase Behavior in Thin Films. Nanomaterials.

[49] Daughton J.W. (1982). An Investigation of the Thickness Variation of Spun-on Thin Films Commonly Associated with the Semiconductor Industry. J. Electrochem. Soc..

[50] Jarząbek B., Nitschke P., Hajduk B., Domański M., Bednarski H. (2020). In situ thermo-optical studies of polymer:fullerene blend films. Polym. Test..

[51] Jarząbek B., Nitschke P., Godzierz M., Palewicz M., Piasecki T., Gotszalk T.P. (2022). Thermo-Optical and Structural Studies of Iodine-Doped Polymer: Fullerene Blend Films, Used in Photovoltaic Structures. Polymers.

[52] Clark J., Chang J.F., Spano F.C., Friend R.H., Silva C. (2009). Determining exciton bandwidth and film microstructure in polythiophene films using linear absorption spectroscopy. Appl. Phys. Lett..

[53] Spano F.C. (2006). Absorption in regio-regular poly(3-hexyl)thiophene thin films: Fermi resonances, interband coupling and disorder. Chem. Phys..

[54] Spano F.C. (2005). Modeling disorder in polymer aggregates: The optical spectroscopy of regioregular poly(3-hexylthiophene) thin films. J. Chem. Phys..

[55] Stöcker T., Köhler A., Moos R. (2012). Why Does the Electrical Conductivity in PEDOT:PSS Decrease with PSS Content? A Study Combining Thermoelectric Measurements with Impedance Spectroscopy. J. Polym. Sci. Part B Polym. Phys..

[56] Estrada R.H.C., Folkes M.J. (2002). Structure formation and modelling of the electrical conductivity in SBS-polyaniline blends. J. Mater. Sci. Lett..

