# Supplementary material for: Nanostructure-Dependent Electrical Conductivity Model Within the Framework of the Generalized Effective Medium Theory Applied to Poly(3-hexyl)thiophene Thin Films
Source: Polymers (Basel). 2024 Nov 20;16(22):3227. doi: 10.3390/polym16223227 (PMC11598009; doi:10.3390/polym16223227)
Supplement: Supplementary file 1 [file polymers-16-03227-s001.zip › polymers-3271609-supplementary-final/Supplementary Materials HB.v8.pdf]

Supplementary Materials

Nanostructure-Dependent Electrical Conductivity Model Within the Framework of the Generalized Effective Medium Theory Applied to Poly(3-hexyl)thiophene Thin Films

Henryk Bednarski [1,*], Ayman A. A. Ismail [1,2], Marcin Godzierz [1], Andrzej Marcinkowski [1], Muhammad Raheel Khan [1,2], Bożena Jarząbek [1], Barbara Hajduk [1] and Pallavi Kumari [1]

[1] Centre of Polymer and Carbon Materials, Polish Academy of Sciences, ul. M. Curie-Skłodowskiej 34, 41-819 Zabrze, Poland
[2] Joint Doctoral School, Silesian University of Technology, Akademicka 2a, 44-100 Gliwice, Poland
* Correspondence: hbednarski@cmpw-pan.pl

Contents:

1. Summary of films preparation conditions

2. XRD patterns for films deposited from 10 and 30 mg/ml solutions concentration; Figures 1S

3. 3D surface AFM images of P3HT films; Figures 2S and 3S

4. Surface roughness parameter Rq, as a function of spin coating speed; Figure 4S

5. Transmittance of studied P3HT films; Figures 5S.

6. Normalized absorption spectra of studied P3HT films; Figures 6S

7. Details of the number of molecules, N, forming the aggregate determination

1. Summary of films preparation conditions

Table S1. Summary of films preparation conditions.

**Solution Concentration**	**Substrate Type**	**Spin Speeds (rpm)**	**Spin Time**	**Spinning rea**	**Initial Heating**	**Electrode Application**	**Final Heating**
20 mg/mL	Glass	750,1000<br>1250,1500<br>3000,4500	60 s	3 s	120°C, 5 min	Silver paste	65°C, 5 min
40 mg/ml	Glass	1250,1500<br>1575,1725<br>2000,3500,<br>5000,7000	60 s	3 s	120°C, 5 min	Silver paste	65°C, 5 min
30 mg/ml	Glass	750,1000<br>1500,1750<br>2000,3000	60 s	3 s	120°C, 5 min	_____	_____
10 mg/ml	Glass	500,750<br>1000,1500<br>2000	60 s	3 s	120°C, 5 min	_____	_____

2. XRD patterns for films deposited from solutions with concentrations of 10 and 30 mg/ml

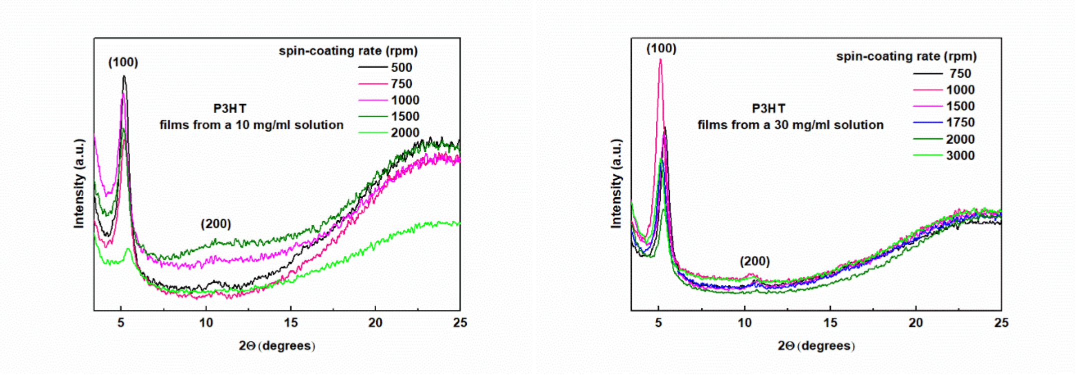

(a) (b)

Figure S1. XRD patterns of P3HT films deposited from solutions with concentrations: (a) of 10 mg/mL and (b) 30 mg/mL solution concentration with indicated spin-coating rates.

3. 3D surface AFM images of P3HT films deposited with different spin-coating rates

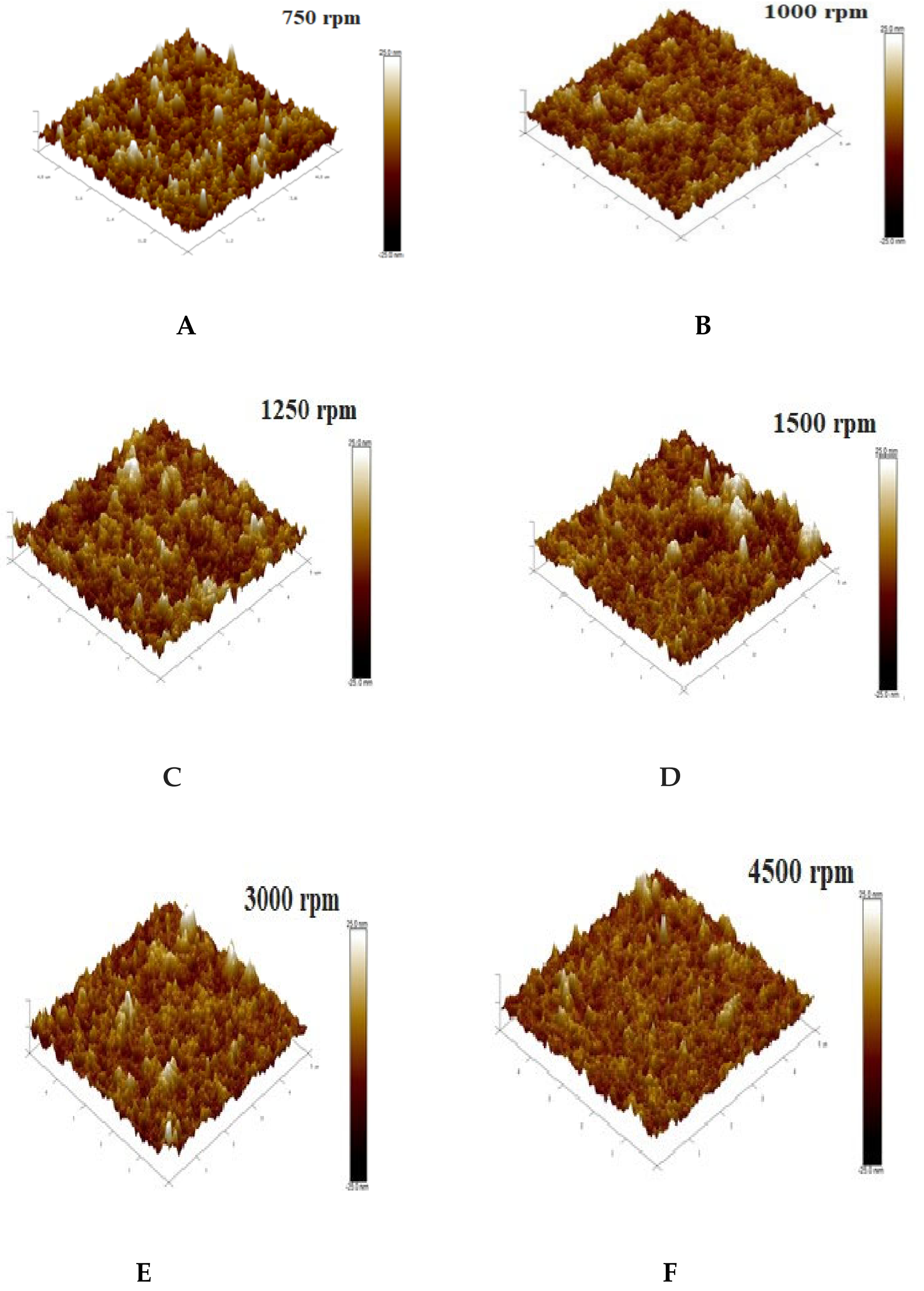

Figure S2. 3D surface AFM images of P3HT films deposited with indicated spin-coating rates from 20 mg/ml solution.

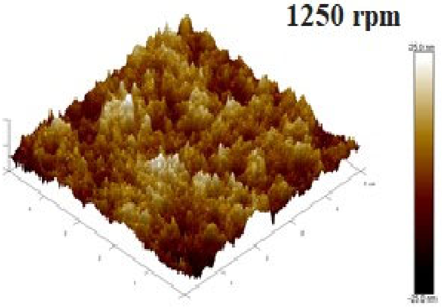

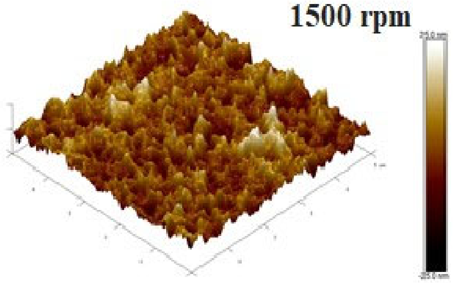

A

B

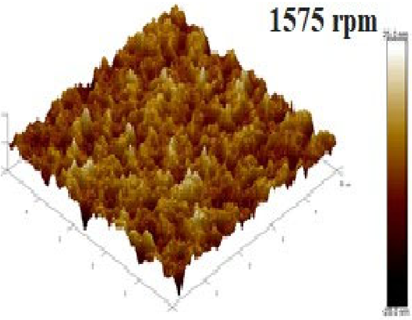

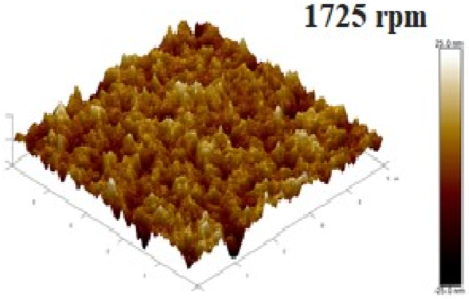

C

D

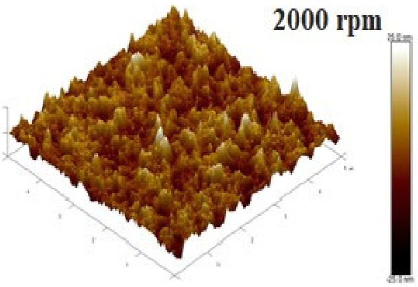

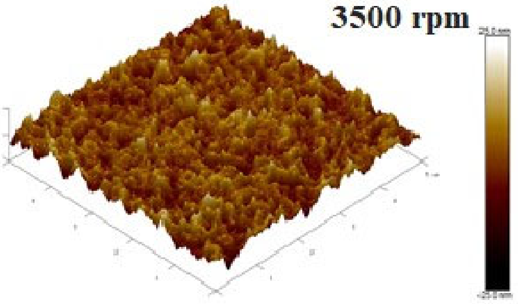

E

F

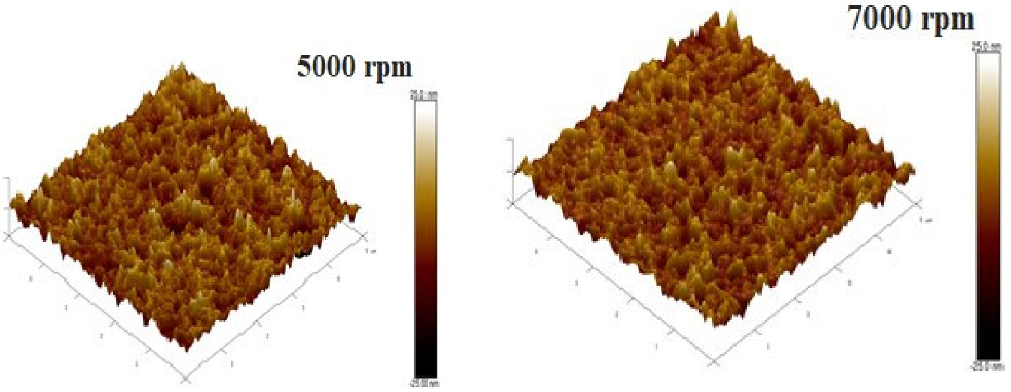

G **H**

Figure S3. 3D surface AFM images of P3HT films deposited with indicated spin-coating rates from 40 mg/ml solution.

4. Results from AFM surface images analysis

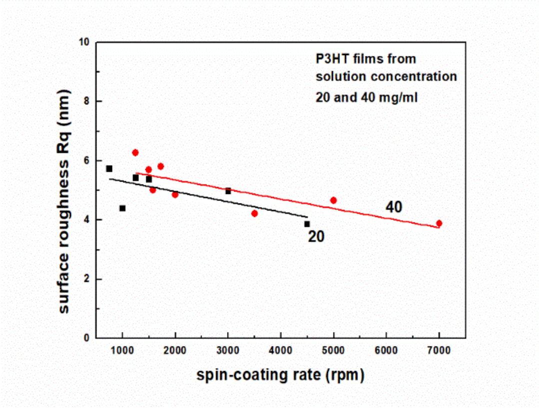

Figure S4. Surface roughness parameter Rq, as a function of spin coating rate.

5. Raw data from optical transmittance measurements

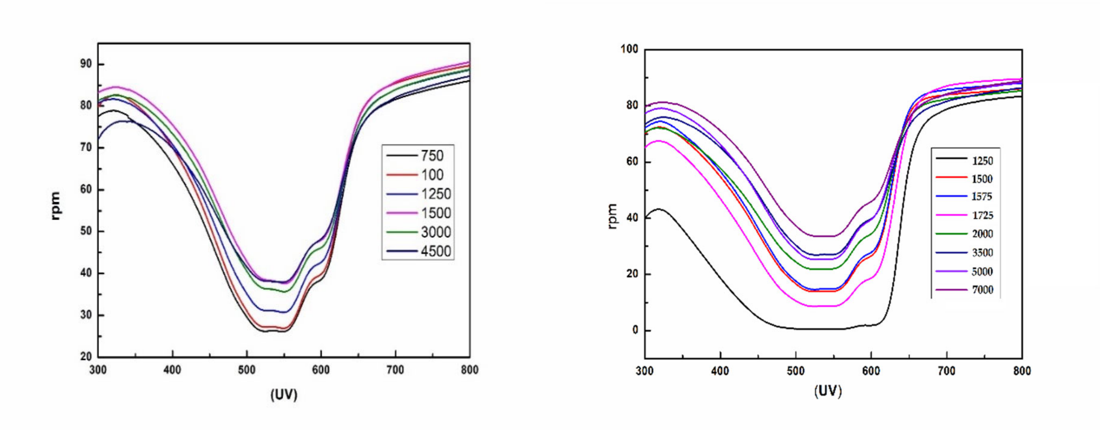

(a) (b)

Figure S5. The transmittance of the studied films deposited from solutions with concentrations of: (a) 20 mg/mL and (b) 40 mg/mL.

6. Normalized absorption spectra of studied P3HT films

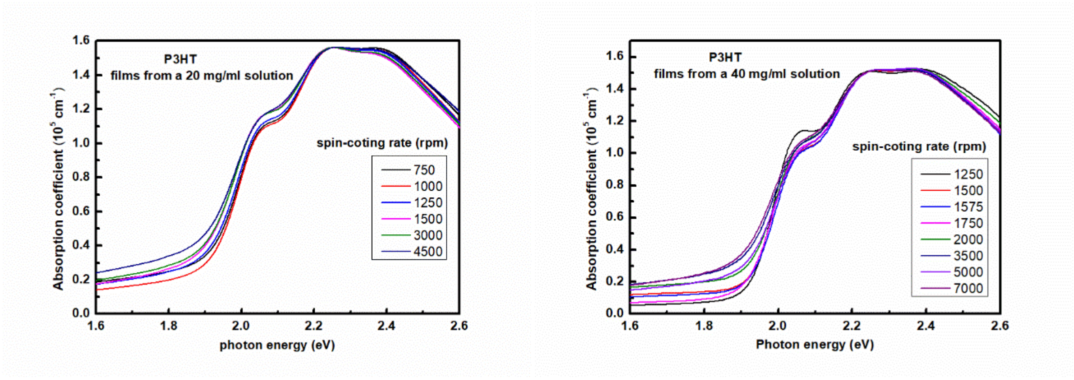

(a) (b)

Figure S6. Normalized absorption spectra of P3HT thin films deposited from: (a) 20 mg/mL and (b) 40 mg/mL solution concentration with indicated spin-coating rates.

7. Details of the number of molecules, N, forming the aggregate determination

$$\left(\frac{0.106}{0.116}\right)^2 = 0.835 \qquad \frac{25+1}{30+1} = 0.839$$

$$\left(\frac{0.106}{0.117}\right)^2 = 0.821 \qquad \frac{24.5+1}{30+1} = 0.823$$

$$\left(\frac{0.106}{0.118}\right)^2 = 0.807 \qquad \frac{24+1}{30+1} = 0.806$$

$$\left(\frac{0.106}{0.112}\right)^2 = 0.896 \qquad \frac{27+1}{30+1} = 0.903$$

$$\left(\frac{0.116}{0.118}\right)^2 = 0.966 \qquad \frac{24+1}{25+1} = 0.962$$

$$\left(\frac{0.112}{0.118}\right)^2 = 0.901 \qquad \frac{24+1}{27+1} = 0.893$$

$$\left(\frac{0.106}{0.12}\right)^2 = 0.78 \qquad \frac{23+1}{30+1} = 0.774$$

$$\left(\frac{0.113}{0.115}\right)^2 = 0.966 \qquad \frac{25+1}{26+1} = 0.963$$

$$\left(\frac{0.113}{0.117}\right)^2 = 0.933 \qquad \frac{24.5+1}{26+1} = 0.944$$

$$\left(\frac{0.113}{0.124}\right)^2 = 0.83 \qquad \frac{21+1}{26+1} = 0.815$$

$$\left(\frac{0.113}{0.118}\right)^2 = 0.917 \qquad \frac{24+1}{26+1} = 0.926$$

$$0.106 \cdot \frac{1}{\sqrt{\frac{3}{2\cdot(30+1)}}} = 0.482$$
