# Supplementary figures and images for: Nanostructure-Dependent Electrical Conductivity Model Within the Framework of the Generalized Effective Medium Theory Applied to Poly(3-hexyl)thiophene Thin Films
Source: Polymers (Basel). 2024 Nov 20;16(22):3227. doi: 10.3390/polym16223227 (PMC11598009; doi:10.3390/polym16223227)

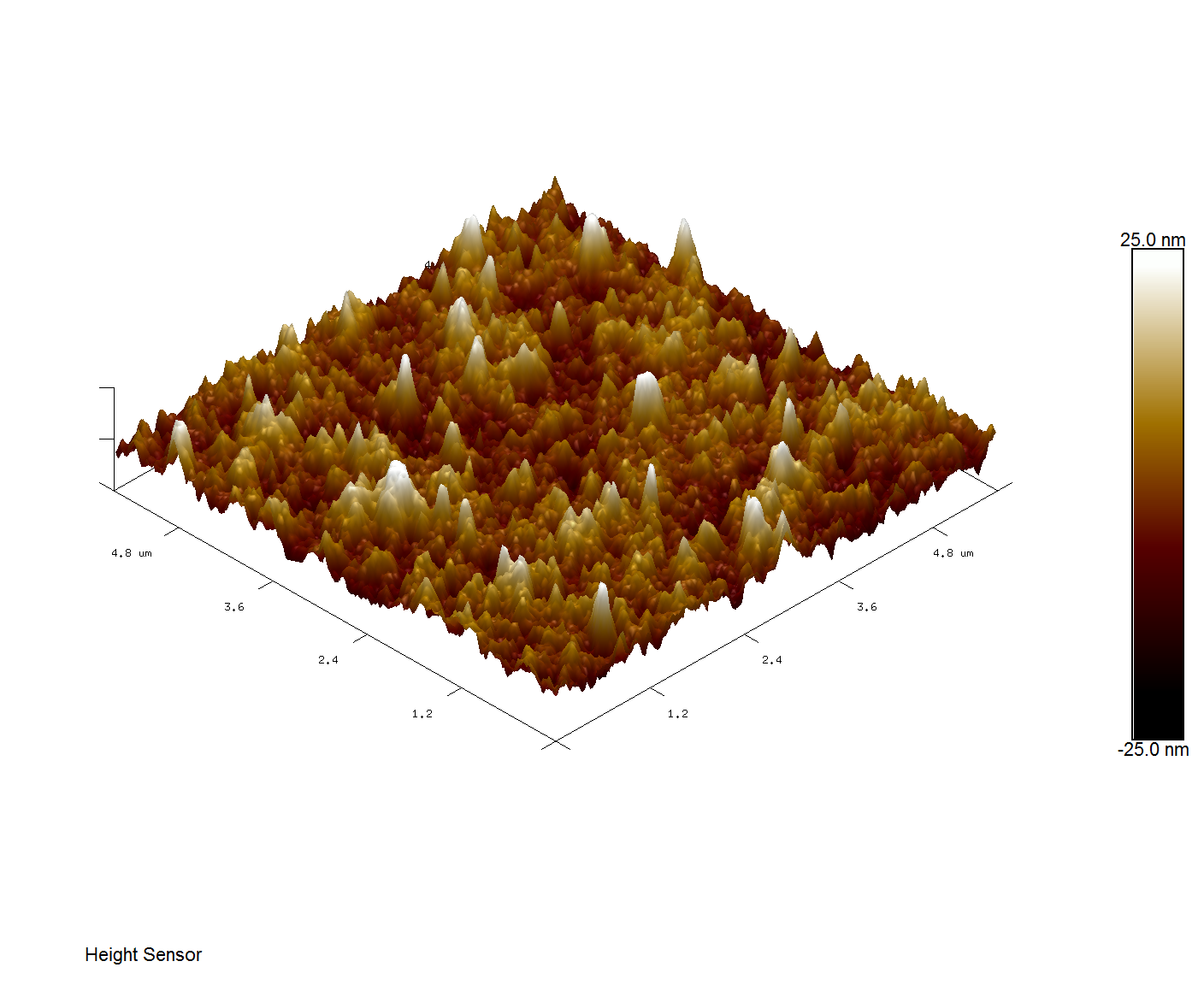

Supplement: Supplementary file 1 [file polymers-16-03227-s001.zip › polymers-3271609-supplementary-final/Figure_S2a.tif]

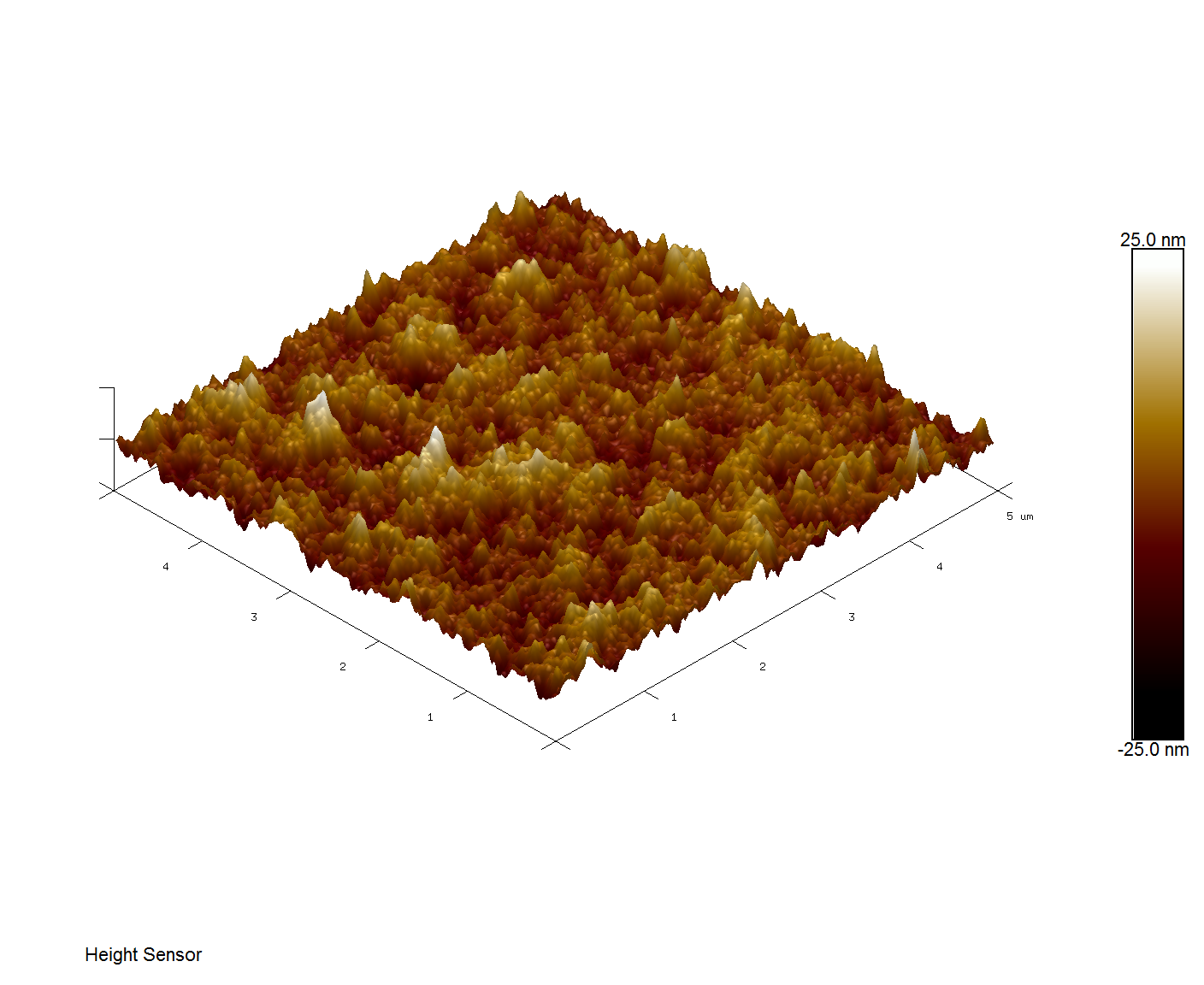

Supplement: Supplementary file 1 [file polymers-16-03227-s001.zip › polymers-3271609-supplementary-final/Figure_S2b.tif]

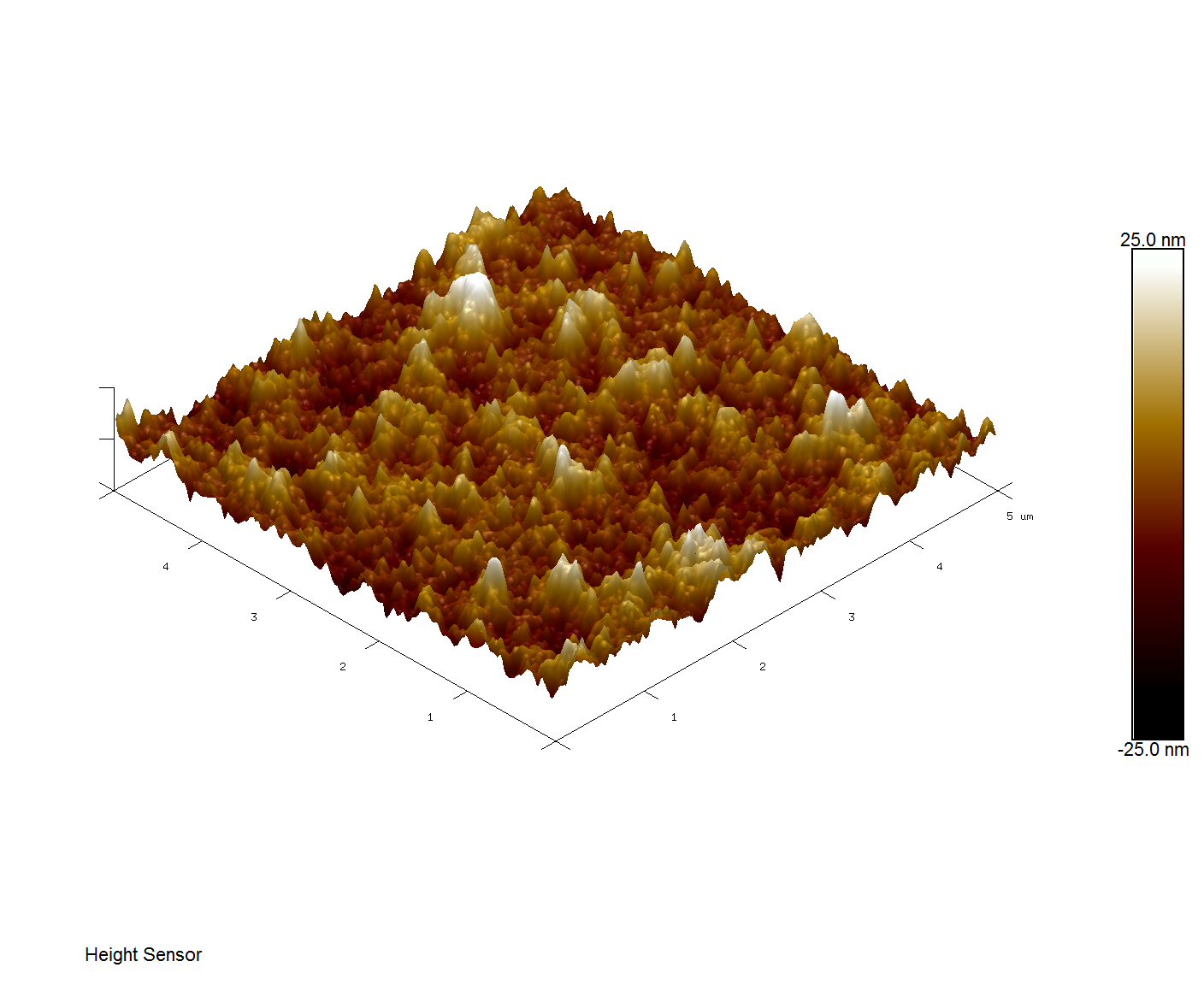

Supplement: Supplementary file 1 [file polymers-16-03227-s001.zip › polymers-3271609-supplementary-final/Figure_S2c.tif]

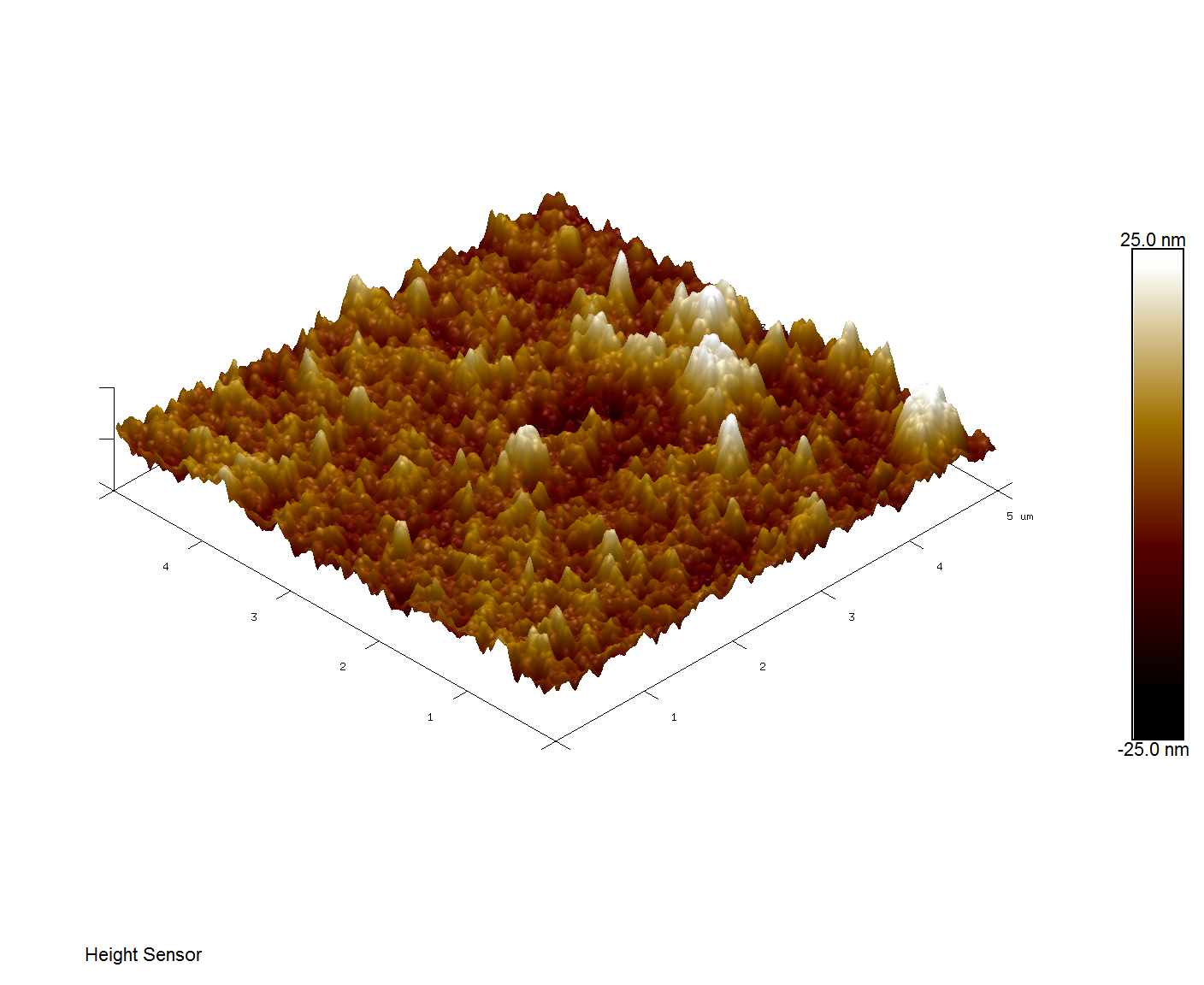

Supplement: Supplementary file 1 [file polymers-16-03227-s001.zip › polymers-3271609-supplementary-final/Figure_S2d.tif]

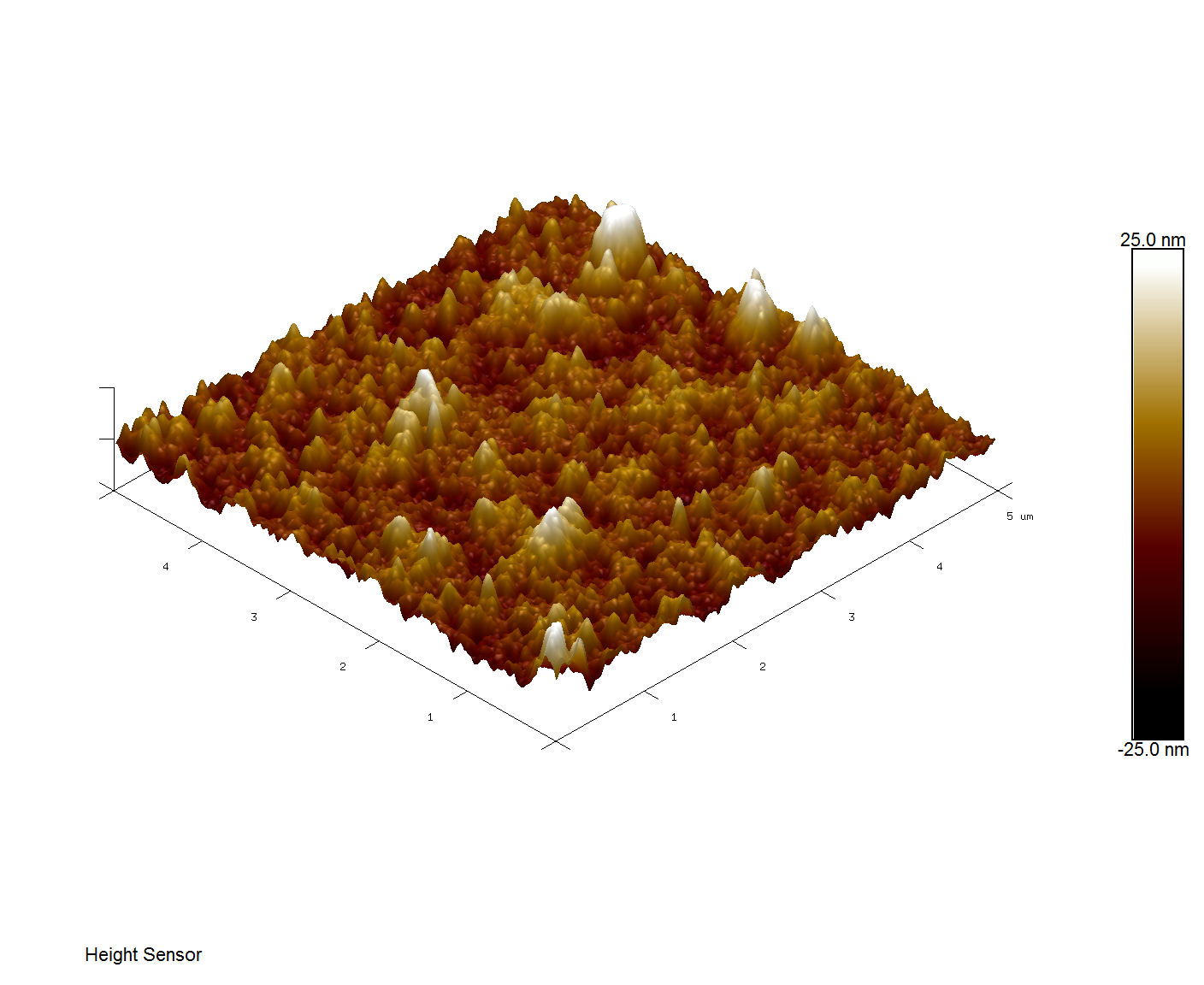

Supplement: Supplementary file 1 [file polymers-16-03227-s001.zip › polymers-3271609-supplementary-final/Figure_S2e.tif]

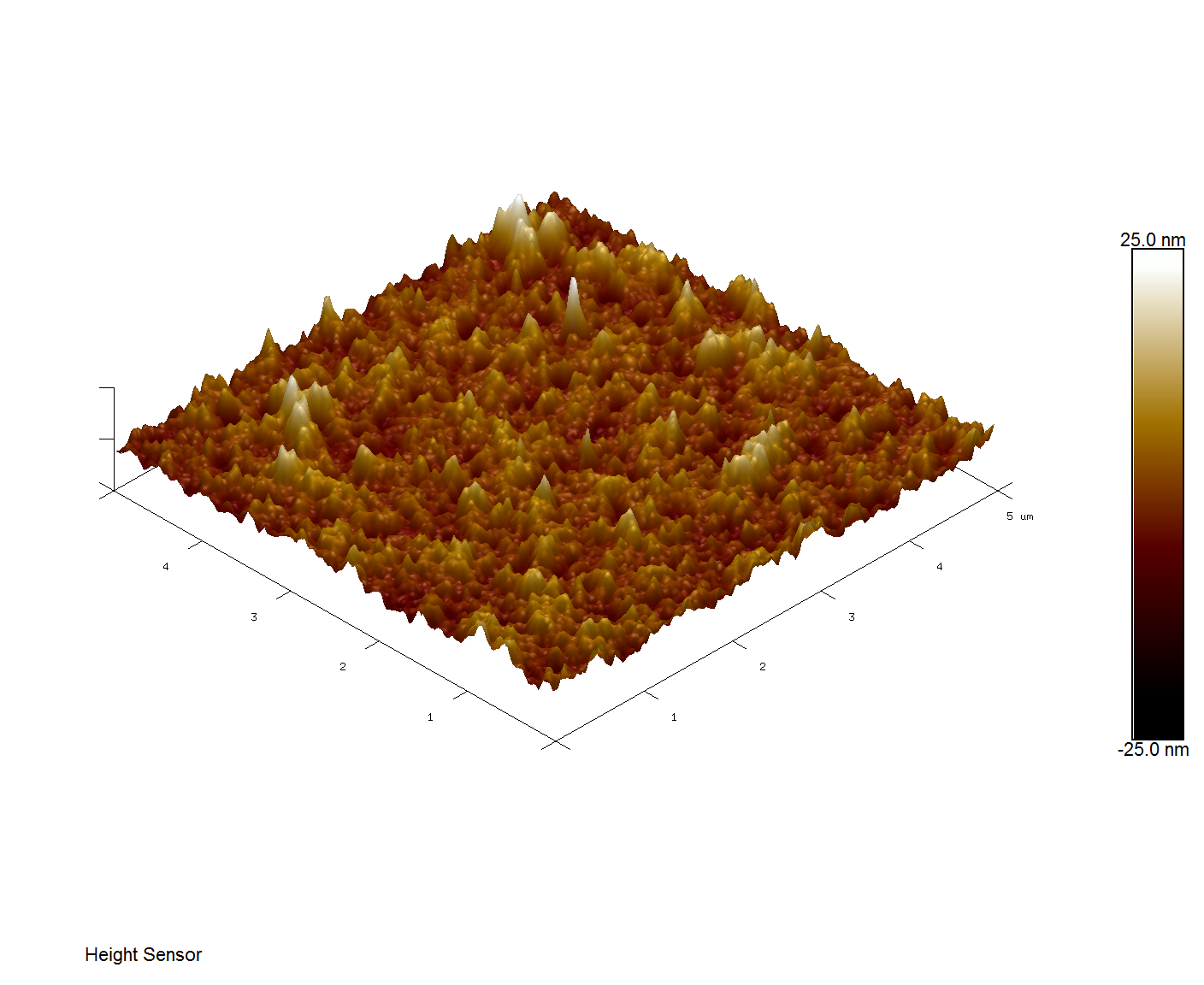

Supplement: Supplementary file 1 [file polymers-16-03227-s001.zip › polymers-3271609-supplementary-final/Figure_S2f.tif]

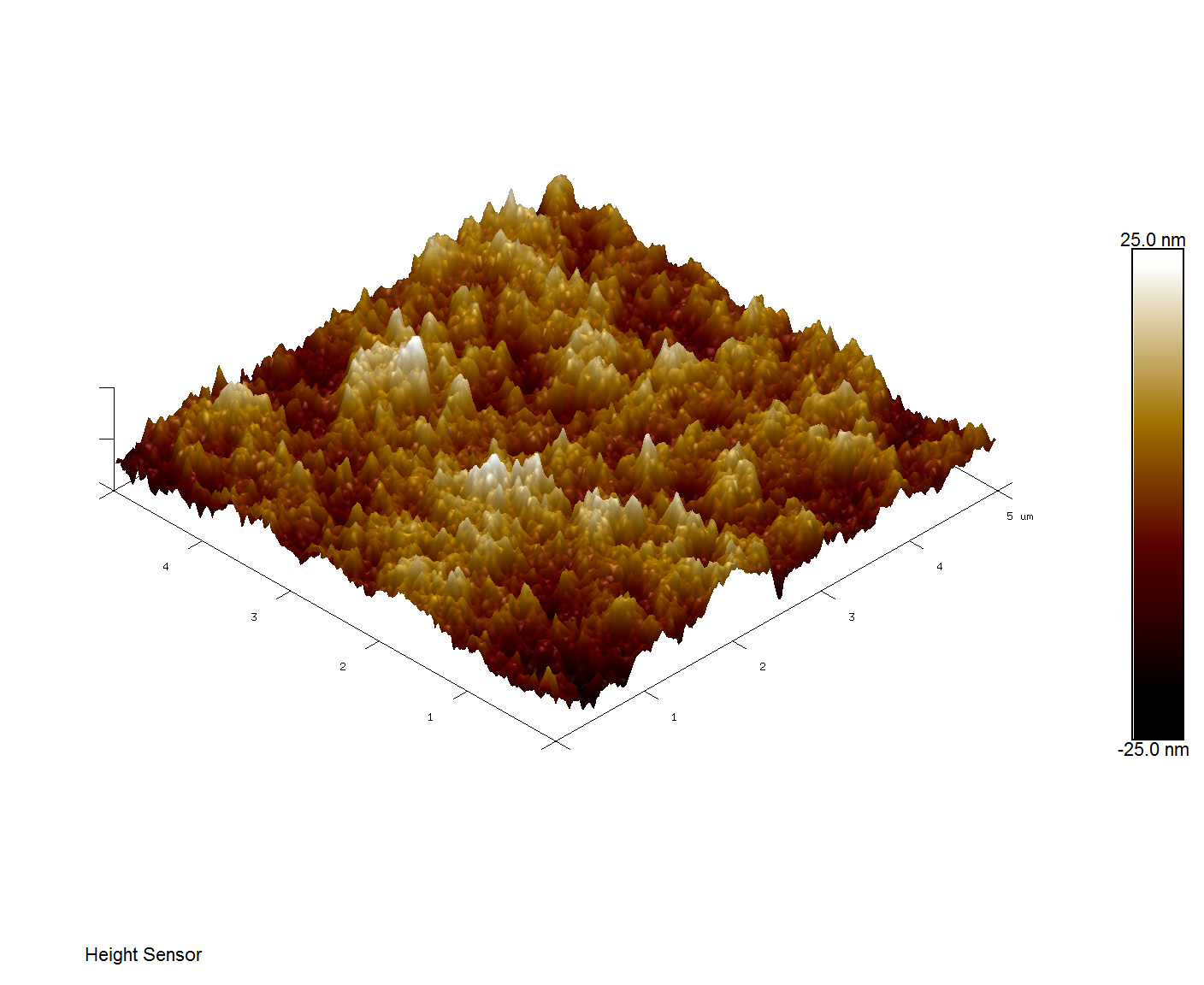

Supplement: Supplementary file 1 [file polymers-16-03227-s001.zip › polymers-3271609-supplementary-final/Figure_S3a.tif]

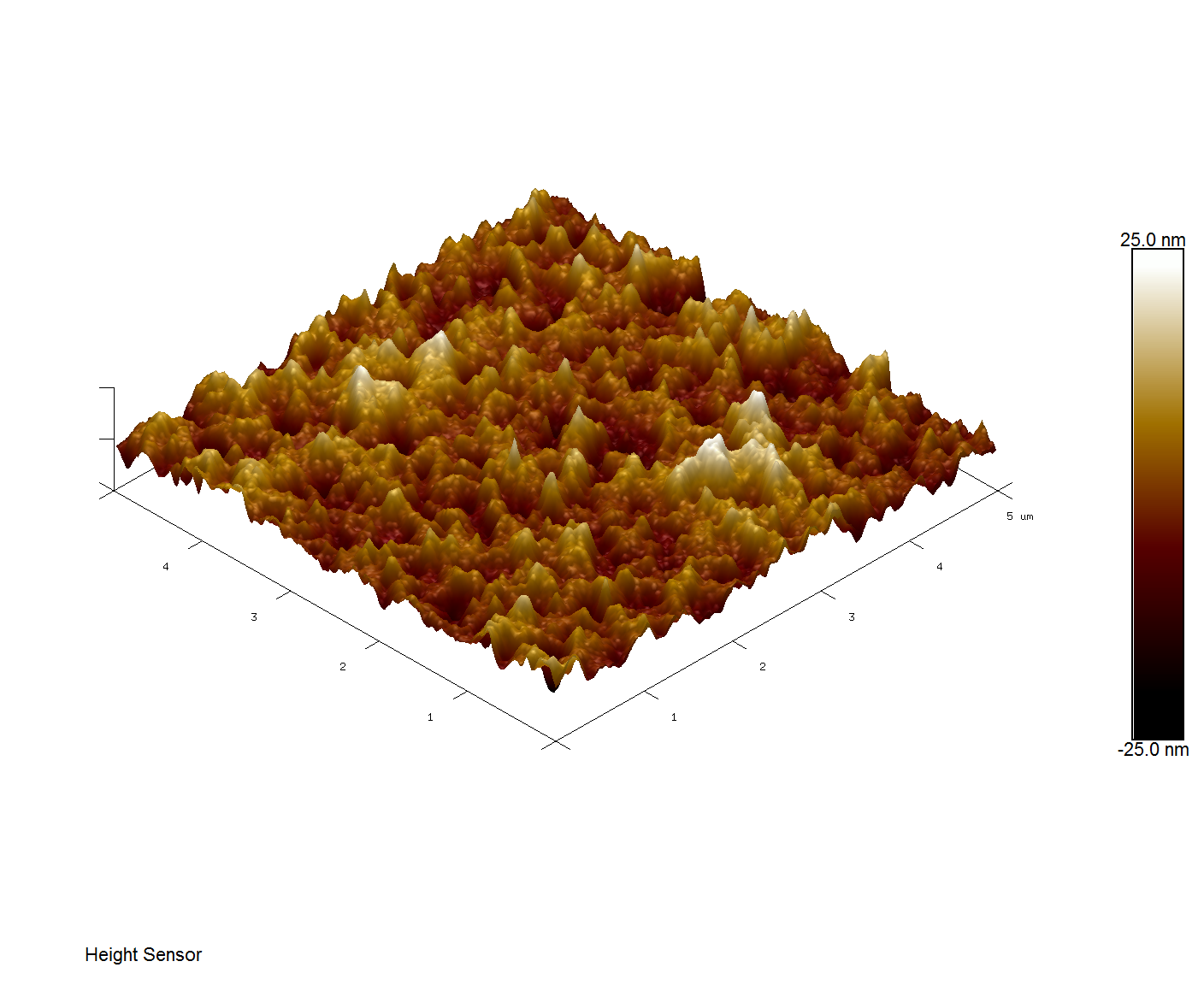

Supplement: Supplementary file 1 [file polymers-16-03227-s001.zip › polymers-3271609-supplementary-final/Figure_S3b.tif]

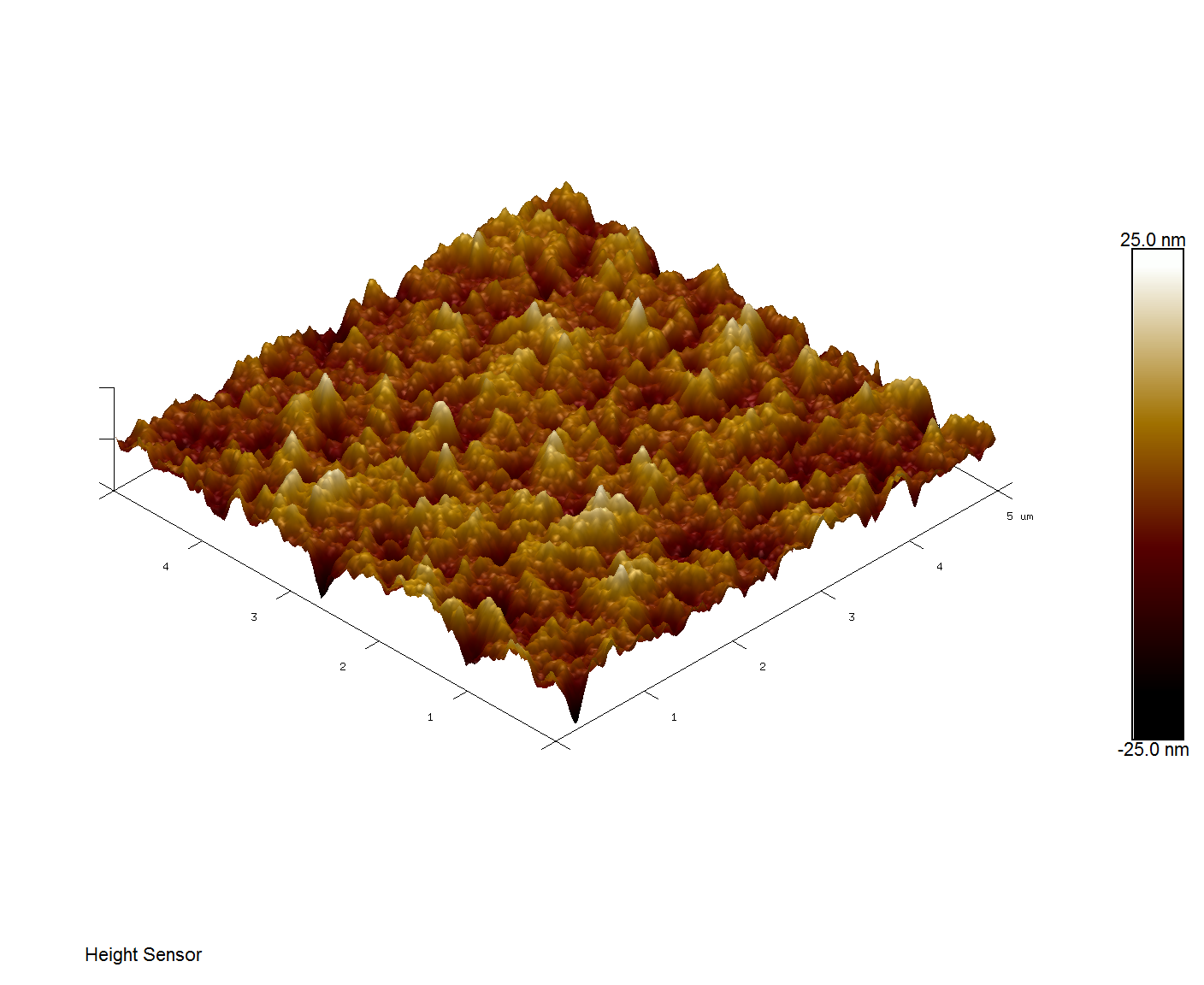

Supplement: Supplementary file 1 [file polymers-16-03227-s001.zip › polymers-3271609-supplementary-final/Figure_S3c.tif]

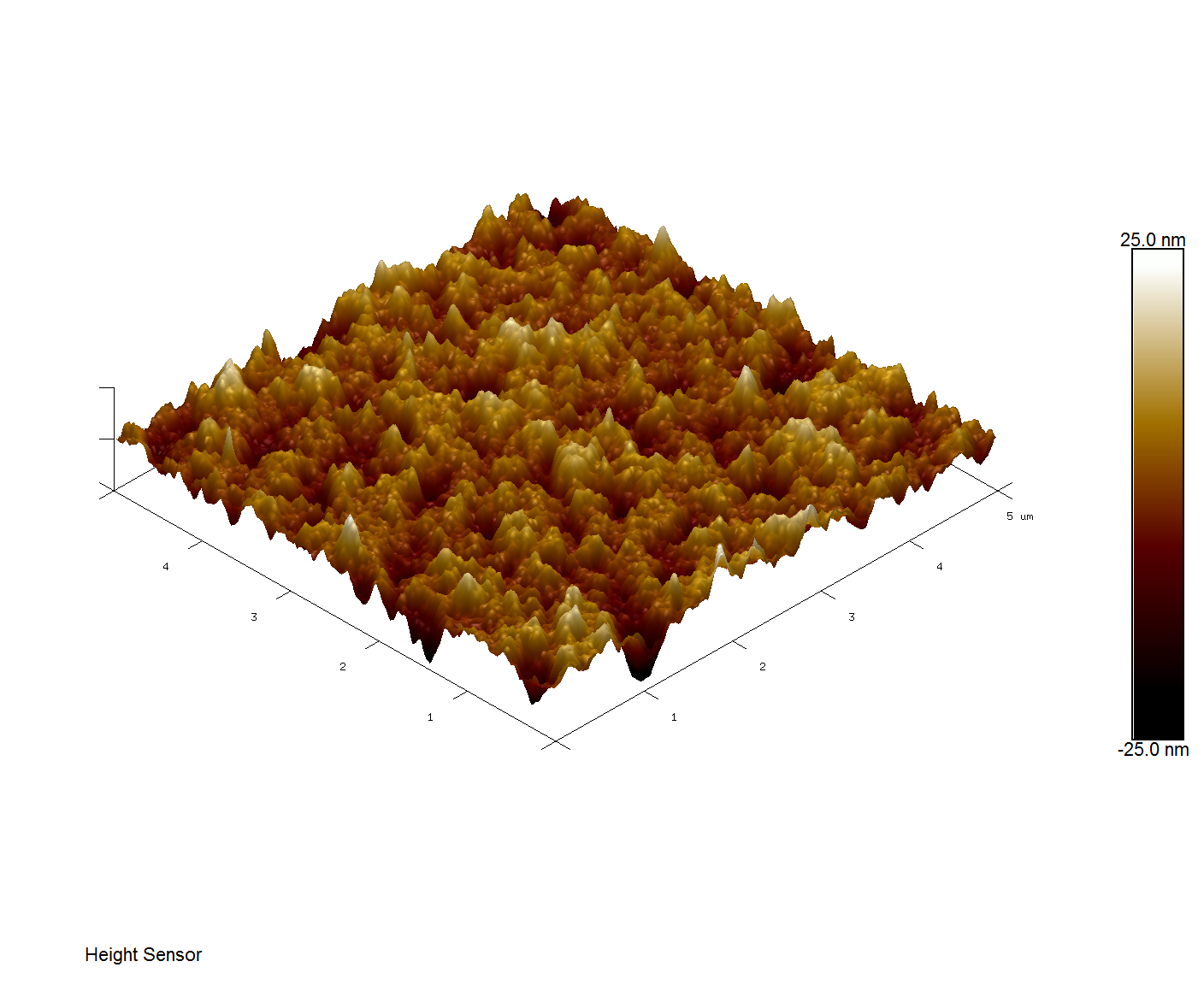

Supplement: Supplementary file 1 [file polymers-16-03227-s001.zip › polymers-3271609-supplementary-final/Figure_S3d.tif]

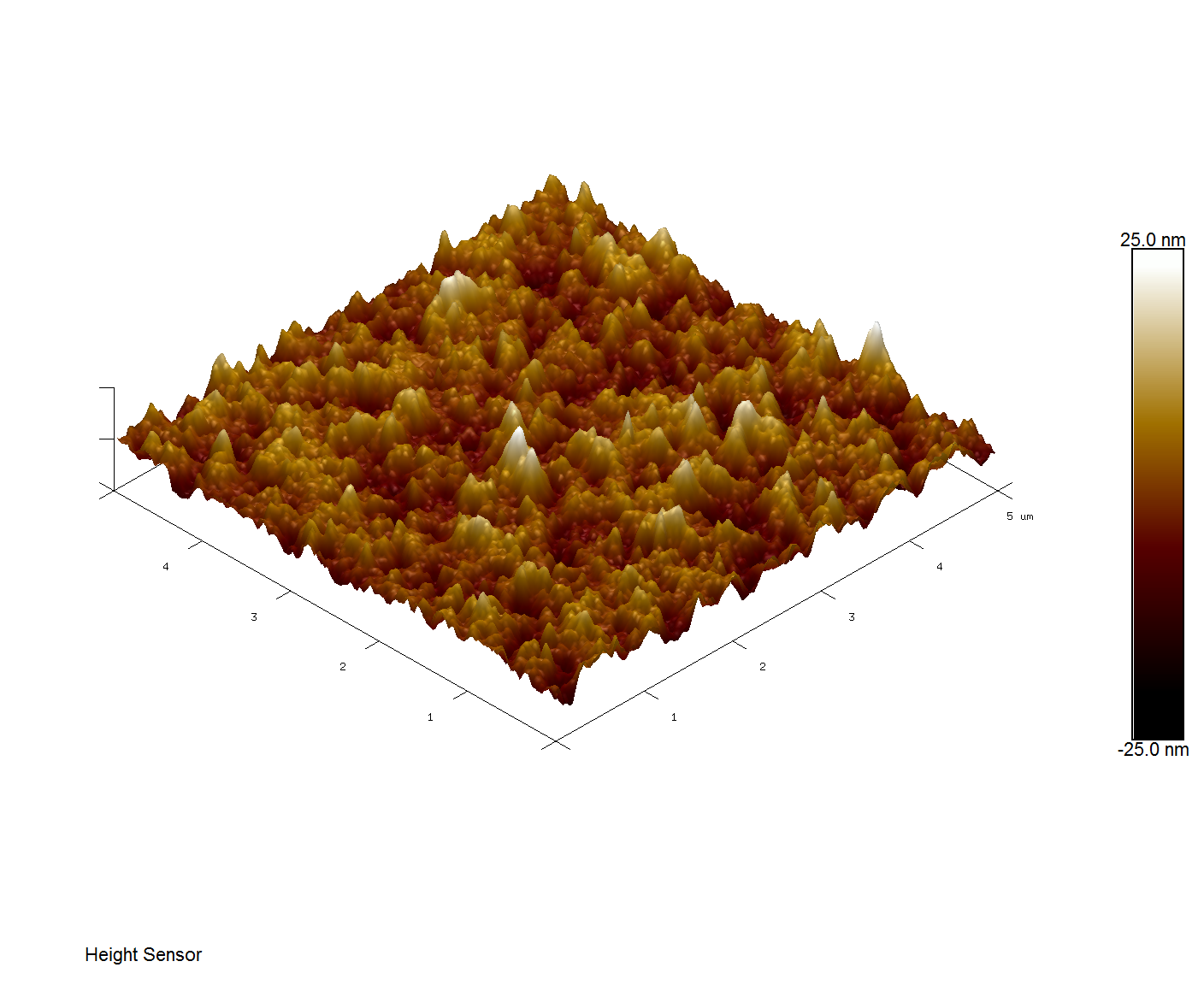

Supplement: Supplementary file 1 [file polymers-16-03227-s001.zip › polymers-3271609-supplementary-final/Figure_S3e.tif]

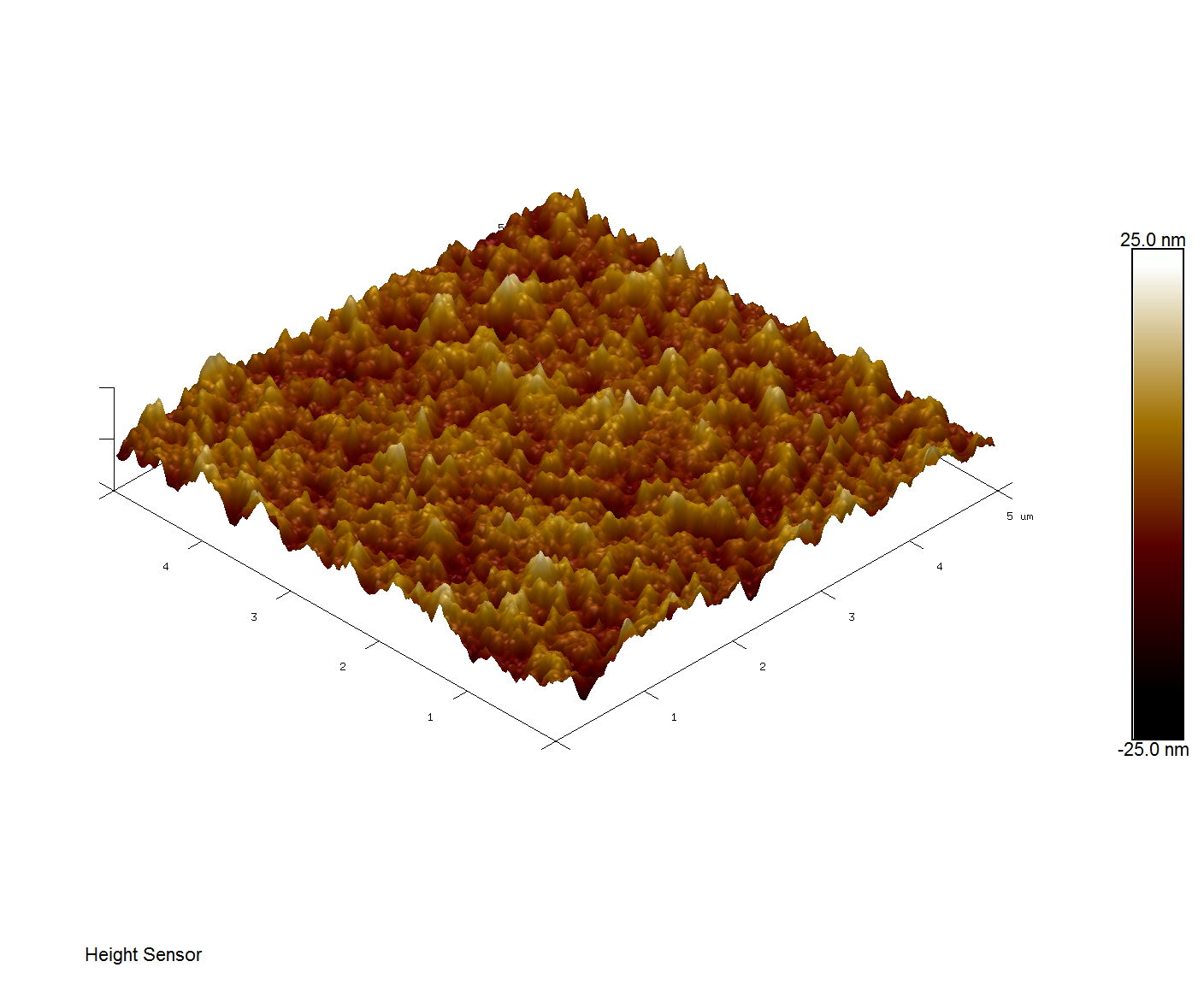

Supplement: Supplementary file 1 [file polymers-16-03227-s001.zip › polymers-3271609-supplementary-final/Figure_S3f.tif]

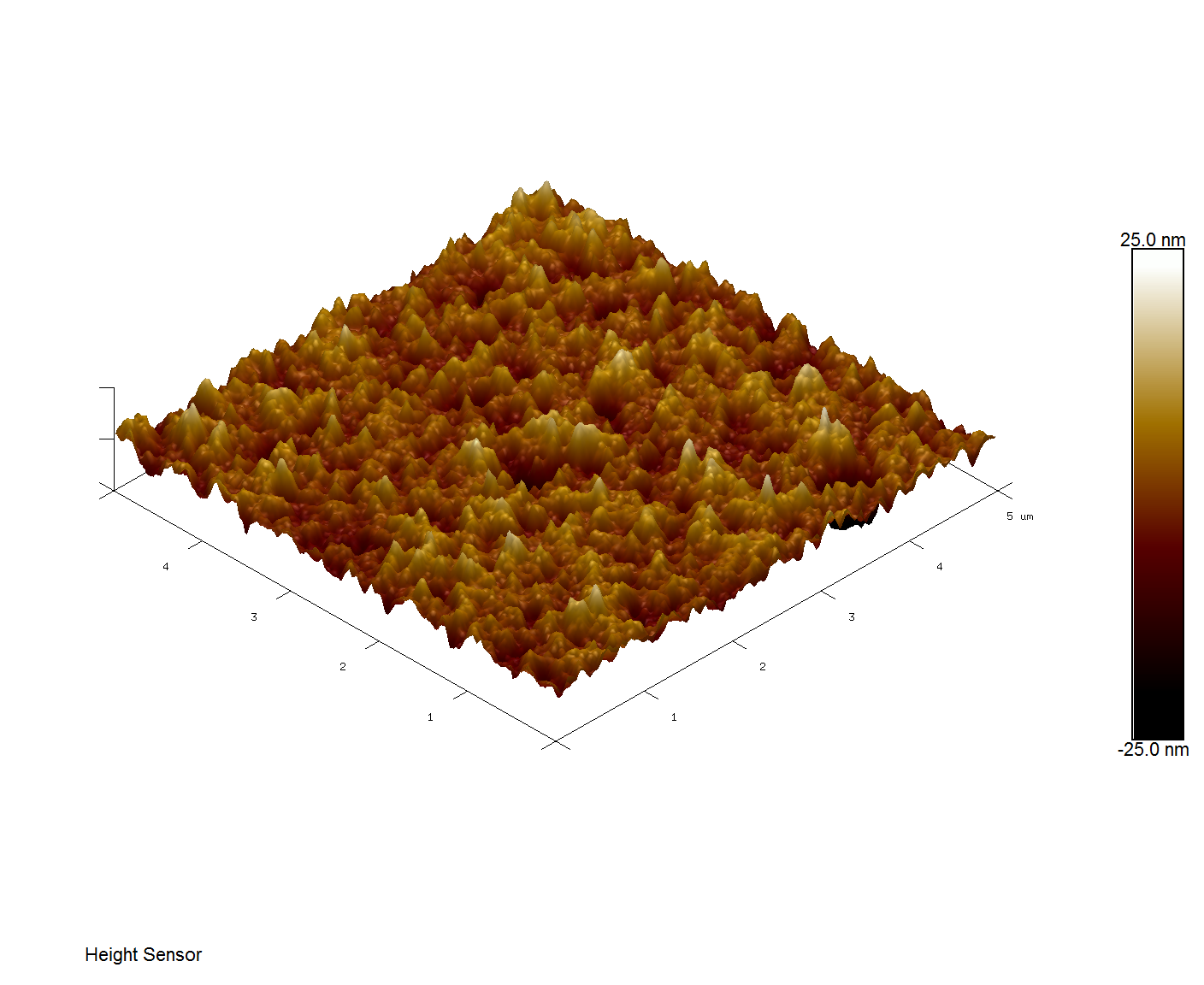

Supplement: Supplementary file 1 [file polymers-16-03227-s001.zip › polymers-3271609-supplementary-final/Figure_S3g.tif]

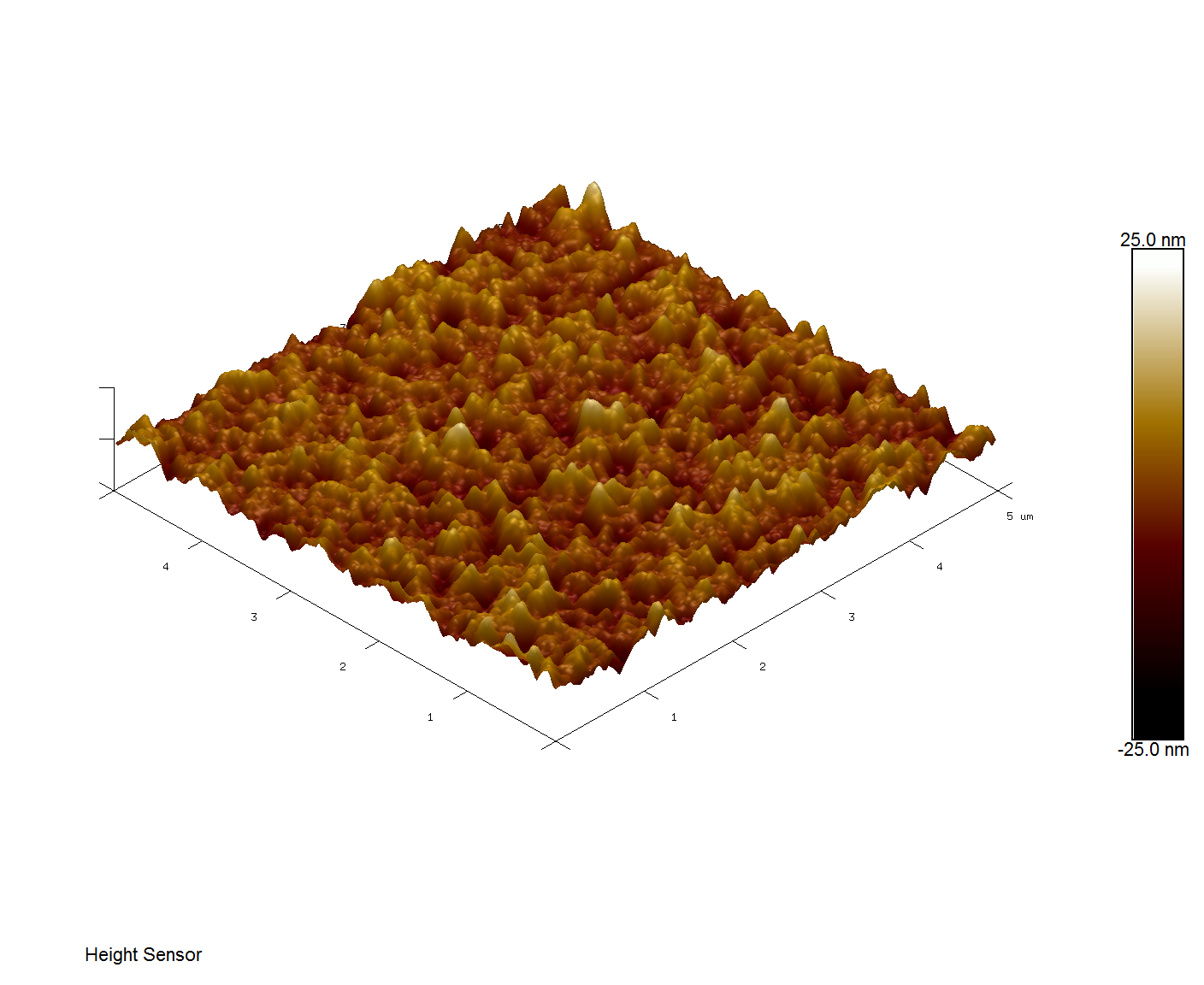

Supplement: Supplementary file 1 [file polymers-16-03227-s001.zip › polymers-3271609-supplementary-final/Figure_S3h.tif]

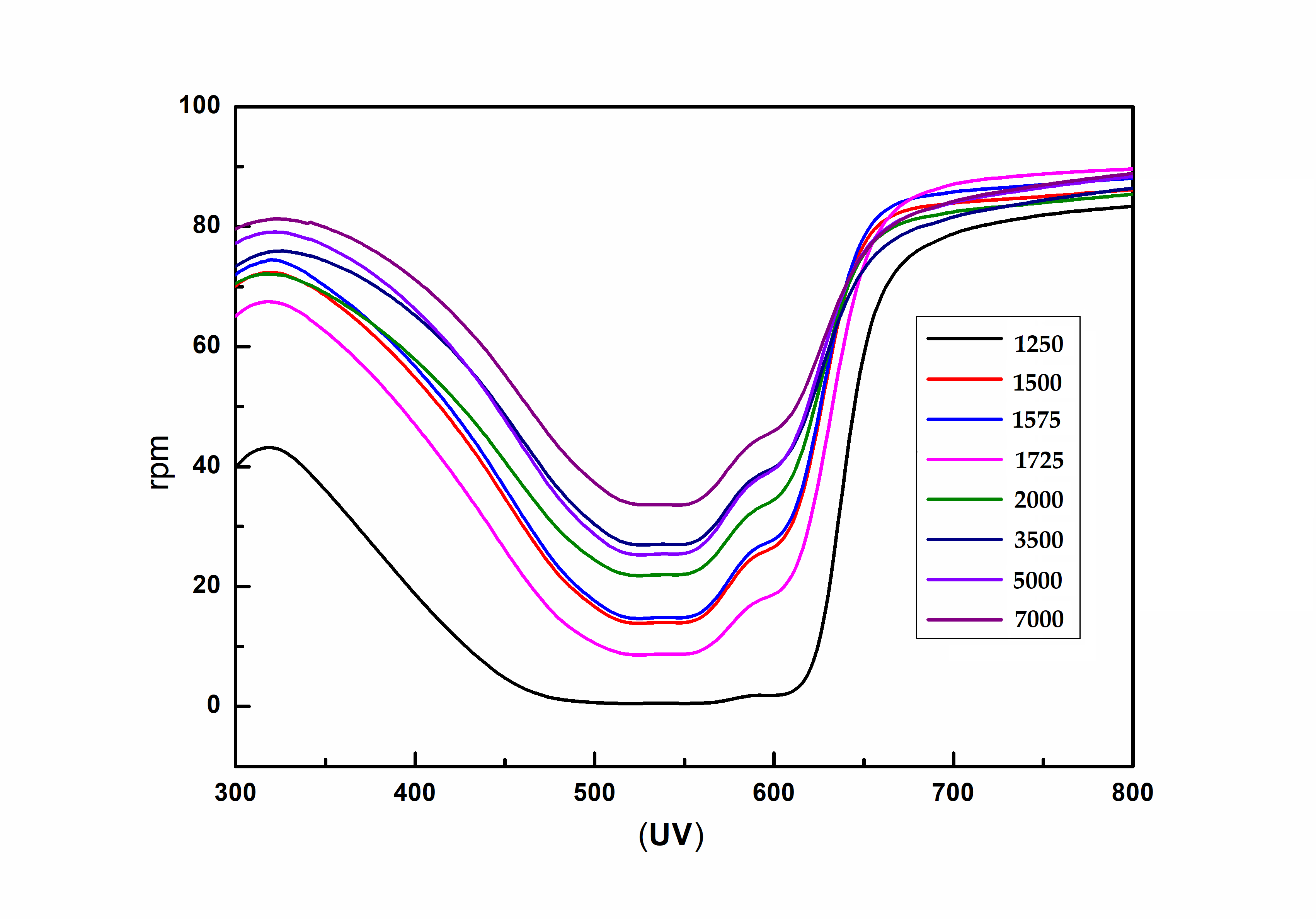

Supplement: Supplementary file 1 [file polymers-16-03227-s001.zip › polymers-3271609-supplementary-final/Figure_S5b.tif]
